# Respiratory 4D-Gating F-18 FDG PET/CT Scan for Liver Malignancies: Feasibility in Liver Cancer Patient and Tumor Quantitative Analysis

**DOI:** 10.3389/fonc.2022.789506

**Published:** 2022-02-09

**Authors:** Anson H. Y. Cheung, Vincent W. C. Wu, Andy L. Y. Cheung, Jing Cai

**Affiliations:** ^1^ Department of Health Technology & Informatics, The Hong Kong Polytechnic University, Hong Kong, Hong Kong SAR, China; ^2^ Radiotherapy and Oncology Department, Hong Kong Baptist Hospital, Hong Kong, Hong Kong SAR, China; ^3^ Department of Clinical Oncology, Queen Mary Hospital, Hong Kong, Hong Kong SAR, China

**Keywords:** liver, 4D PET/CT, respiratory gated PET/CT, SUV, clinical protocol

## Abstract

**Purpose:**

To evaluate the potential clinical role and effectiveness of respiratory 4D-gating F-18 FDG PET/CT scan for liver malignancies, relative to routine (3D) F-18 FDG PET/CT scan.

**Materials and Methods:**

This study presented a prospective clinical study of 16 patients who received F-18 FDG PET/CT scan for known or suspected malignant liver lesions. Ethics approvals were obtained from the ethics committees of the Hong Kong Baptist Hospital and The Hong Kong Polytechnic University. Liver lesions were compared between the gated and ungated image sets, in terms of 1) volume measurement of PET image, 2) accuracy of maximum standardized uptake value (SUV_max_), mean standardized uptake value (SUV_mean_), and 3) accuracy of total lesion glycoses (TLG). Statistical analysis was performed by using a two-tailed paired Student *t*-test and Pearson correlation test.

**Results:**

The study population consisted of 16 patients (9 males and 7 females; mean age of 65) with a total number of 89 lesions. The SUV_max_ and SUV_mean_ measurement of the gated PET images was more accurate than that of the ungated PET images, compared to the static reference images. An average of 21.48% (p < 0.001) reduction of the tumor volume was also observed. The SUV_max_ and SUV_mean_ of the gated PET images were improved by 19.81% (p < 0.001) and 25.53% (p < 0.001), compared to the ungated PET images.

**Conclusions:**

We have demonstrated the feasibility of implementing 4D PET/CT scan for liver malignancies in a prospective clinical study. The 4D PET/CT scan for liver malignancies could improve the quality of PET image by improving the SUV accuracy of the lesions and reducing image blurring. The improved accuracy in the classification and identification of liver tumors with 4D PET image would potentially lead to its increased utilization in target delineation of GTV, ITV, and PTV for liver radiotherapy treatment planning in the future.

## Introduction

Positron emission tomography/computed tomography (PET/CT) has been proven irreplaceable in providing anatomical and functional radiological information. Fluorodeoxyglucose fluorine-18 (F-18 FDG) PET/CT has been widely utilized for modern oncology imaging and considered as a useful tool for disease staging, assessment of patients’ response to drug therapy, and detection of local recurrence or metastases (1, 2). Primary and secondary liver malignancies typically show increased glucose uptake and metabolism. Tumors consume FDG as a glucose analogue and display a strong uptake in PET images (1). Studies have shown that the sensitivity of detecting liver malignancies with F-18 FDG PET image ranged from 90% to 95% (3–5). The acquisition of PET/CT images consists of two parts: first, CT data are acquired by scanning the entire patient body in a few seconds. Secondly, PET data are acquired by using PET ring detectors; a range of 6–7 bed position acquisition is typically used for an adult scan, and the acquisition time can be up to several minutes per bed.

Of note, the acquired CT and PET image data are derived from an average of multiple respiratory cycles (6). The potential deleterious impact of respiration-induced motion of the upper thorax on accurate image acquisition and target delineation for radiotherapy has been well-documented in literature (6–10). In general, respiration-induced organ motion during PET/CT image acquisitions may cause four problems: 1) motion artifact in CT images, 2) image misregistration between CT and PET image data, 3) image blurring of PET uptake images, and 4) PET reconstruction error due to CT attenuation error (9, 10). The issues of image artifacts and image blurring result in qualitative and quantitative inaccuracy in determination of tumor morphology and its uptake activity in the attenuation-corrected PET/CT images (9, 10). To improve the accuracy of CT and PET image registration and minimize the issue of image blurring, the respiratory gating method has been introduced. The PET/CT scanner is equipped with a respiratory gating system that enables image data sorting. The acquired CT and PET data are equally separated into different respiratory phases, then each phase data within specific respiratory cycles are used for image reconstruction (10). Furthermore, imaging-based prognostic markers are crucial for patients’ treatment option and survival; metabolic parameters derived using standard uptake values (SUV) or total lesion glycolysis (TLG) may be beneficial for disease staging and risk stratification before surgery and radiotherapy treatment (11, 12).

Despite the recognized issues caused by the respiration-induced motion in thoracic imaging, studies on investigating the impact of patients’ breathing motion in PET/CT scan for liver malignancies, especially in a prospective clinical design, are severely scarce in the body of literature. Indeed, determination of the trajectory of respiration-induced liver motion is one of the major challenges in highly precise liver radiotherapy (13). Several studies have shown that liver tumor motion occurs primarily in the superior–inferior (SI) direction, ranging from 5 to 50 mm (14, 15). This respiration-induced tumor motion had adverse influences on radiation therapy treatment planning and delivery, including inaccurate tumors and normal tissue localization (16–18), dosimetric uncertainty based on a static CT images plan (16), and the requirement of increased planning target volume (PTV) margins, potentially leading to overexposure of the surrounding normal tissues and limiting the maximum allowable dose that should be given to the tumors (19, 20). For instance, Crivellaro et al. retrospectively analyzed standard 3D-PET/CT (i.e., ungated) and liver 4D-PET/CT (i.e., gated) images of 56 patients, hoping to investigate the added diagnostic value of respiratory-gated 4D PET/CT in detecting and characterizing a total of 72 liver lesions (21). They reported that an enhanced confidence of physicians in lesion detection on the gated PET/CT was found in 51.4% of the studied lesions, compared to the ungated PET/CT. Besides, they also demonstrated a significantly higher level of the SUV_max_ value for liver lesions in the gated PET, therefore improving quantitative characterization of the lesions, in comparison to the ungated PET (21). In addition, Michael et al. conducted a retrospective analysis on 149 cancer patients to evaluate the impact of data-driven respiratory gating (DDG) on PET image quality and lesion detection (22). They reported that the issue of image blurring in PET images was significantly lower when DDG (i.e., gated) was used, compared to the PET images without DDG application (i.e., ungated). Besides, boundary of organs, including liver and spleen, was rated significantly sharper on the DDG-gated PET images than those on the ungated PET images (22). These retrospective studies have underlined the importance of 4D-gating PET images in liver lesion detection.

In this study, we attempted to perform a prospective clinical study for evaluating the potential clinical role and effectiveness of respiratory 4D-gating F-18 FDG PET/CT scan specifically for liver malignancies. Comparative analyses of the liver lesions between the gated and ungated PET images were made in aspects of 1) volume measurement of PET image, 2) accuracy of maximum standardized uptake value (SUV_max_) and mean standardized uptake value (SUV_mean_), and 3) accuracy of total lesion glycoses (TLG). The success of this study would not only consolidate evidence in previous retrospective studies but also promote the clinical implementation of 4D-gating FDG PET/CT scans in target delineation for liver radiotherapy treatment planning in the future.

## Materials and Methods

### Study Design and Subject Recruitment

**Figure 1:**
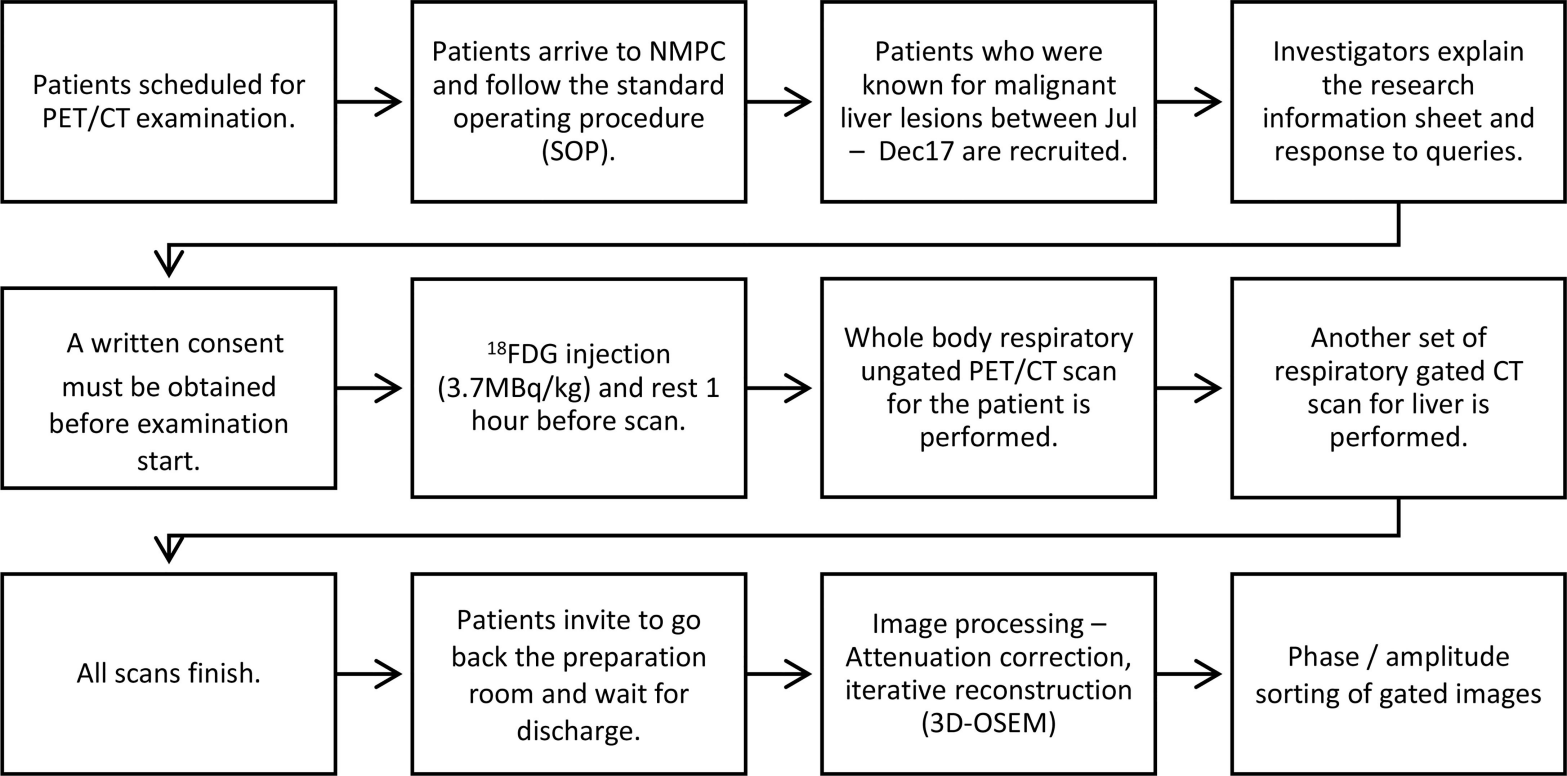
Workflow diagram of patients who were included for non-gated PET/CT and respiratory-gated PET/CT studies.

This study was a prospective study. Ethics approvals were obtained from the ethics committees of the Hong Kong Baptist Hospital and The Hong Kong Polytechnic University. Patients who received F-18 FDG PET/CT scan for known or suspected malignant liver lesions between October 1, 2017, and December 31, 2017, were consecutively recruited in this study. Patients who 1) received previous radiotherapy, 2) were diagnosed with diffused liver lesions on CT images, 3) were diagnosed with benign liver lesions, 4) failed to perform respiratory gated PET/CT scan, or 5) rejected participation of this study were excluded. Verbal and written consents for all subjects were obtained prior to routine scans. The workflow of this study is explained and summarized in **Figure 1**.

### PET/CT Image Acquisitions

All PET/CT scans were carried out on a PET/CT 710 Discovery scanner (GE Healthcare, Milwaukee, WI, USA). This scanner was equipped with time-of-flight (TOF) function, lutetium-based scintillator (LBS) PET scanner, CT detector with 64 rows, and real-time position management respiratory-gated system (RPM, version 1.7.5; Varian Medical Systems, Palo Alto, CA, USA). The RPM motion tracking was set to be three-dimensional as the same as the setup for external beam liver radiotherapy. According to the in-house clinical protocol from the Nuclear Medicine & PET Centre (NMPC), patients were fasted for more than 6 h before FDG injection. The injected dose was related to patients’ body weight with 3.7 MBq per kg, and patients were required to rest for 1 h after injection prior to image acquisition. Patients were instructed to void before getting into the scan room.

The whole-body ungated CT images were acquired under the following imaging parameters: 120 kVp, automatic tube current setting (10–300 mA, noise index 12), CTDI_vol_: 15.68 mGy, 0.938 pitch, 0.4-s speed of gantry rotation, and 3.75-mm thickness per slice. For the routine whole-body scan, images were obtained from the femoral heads to base of skull during a shallow-breathing condition. After whole-body ungated CT scan was obtained, an additional respiratory gating liver CT scan was acquired. The respiratory-gated CT imaging protocol was comparable to those reported by Rietzel et al. (23) and Pan et al. (24). The respiratory-gated CT images for the liver were acquired with the following image parameters: 120 kVp, low-dose mA (10–80 mA, noise index 30), CTDI_vol_: 104.49 mGy, 1.675 pitch, 0.4 s gantry rotation speed, and 2.5 mm slice thickness. It is worth noting that although the CTDIvol for the gated protocol is 6.67-fold higher than that for the non-gated protocol, the maximum tube current was drastically reduced (80 vs. 300 mA) and the pitch was approximately doubled (1.675 vs. 0.938) to reduce CT radiation exposure to the patients in this study. Cine mode in axial slices was performed with continued scans at the liver position with an intervening period equal to 1–1.5 s plus the patient’s mean time of each respiratory cycle. The infrared video camera detected the 3D displacement of two infrared reflective markers in a plastic box which was placed on the patient’s abdomen at the level of the umbilicus. The respiration cycle of the patients was acquired using the RPM system in precise temporal correlation to CT data acquisition.

The whole-body ungated PET images were obtained after the CT scan. A total of 6–7 bed positions were required for a regular adult; all data were collected during shallow breathing for 2 min at each bed position. At the bed position during liver imaging, PET data were acquired for 5 min with respiratory gating. Similar to the respiratory gated CT image acquisition, the RPM system was used to acquire patients’ breathing cycle.

### 4D-Gating and Ungated PET Image Reconstruction

All PET raw data obtained from respiratory gated scan and ungated scan were reconstructed using the 3D Order Subsets Expectation Maximization (3D-OSEM) iterative reconstruction algorithm (24 subsets, 4 iterations) to generate PET images. Details of the image reconstruction parameters are as follows: VUE Point FX reconstructed method, SharpIR quantitation method, 50.0 cm FOV, 192 × 192 matrix, Gaussian 6.0-mm filter of full width at half maximum (FWHM), 3.27-mm thickness per slice, Z-Axis Filter: Standard, and TOF reconstruction algorithm.

### Phantom Validation of the Motion Correction

The QUASAR™ respiratory motion phantom with 4D PET/CT imaging insert (P/N: 500-3318) was used to validate the motion correction before clinical implementation. The PET insert was equipped with a 30-mm sphere which was filled with clinical activity concentrations (injected activity: 3.5 kBq/ml, image acquisition ~ 1 h: ~2.4 kBq/ml) of F-18 FDG (25). The sphere was animated with a 2-cm longitudinal respiratory simulated motion. The movement cycle was set to be 12 breaths per minute. The RPM system was placed on the platform of the QUASAR™ phantom to acquire the movement cycles during data acquisitions. The 4D-gating and ungated PET images were compared to static reference PET images (respiratory motion was disabled).

### Individual Phase Sorting for Respiratory-Gated PET/CT Images

All image data sets were arranged into 10 phases based on the temporal correlation between data acquisition on Advantage Workstation 4.5 (GE Healthcare, Milwaukee, WI, USA) and patients’ breathing displacement motion. The 10 reconstructed respiratory phase images were evenly divided from a full respiratory cycle. Each CT image set was labeled based on their phase of acquisition, e.g., CT_0%, CT_10%, CT_20%, CT_30%, CT_40%, CT_50%, CT_60%, CT_70%, CT_80%, and CT_90%. CT_0% corresponds to end of inspiration, and CT_50% is the start of inspiration or end of expiration.

### Attenuation Correction for Respiratory Gated and Non-Gated PET Images

All CT images were utilized to produce an attenuation correction map that could then be utilized to correct the attenuation effect of 511-keV emission photon passing through the body (26). For PET images which are respiratory gated, phase-matched attenuation correction was carried out using the respiratory-sorted CT images. For PET images obtained from shallow-breathing whole-body scan (ungated scan), they were corrected with the corresponding shallow-breathing CT image set. The gated PET image was based on the registered (i.e., phase-matched) gating image. When the PET/CT examination was acquired based on the mentioned protocol, the examination is defined as successful. Finally, all DICOM CT images were exported to a contouring workstation (MIM Maestro™, MIM Software Inc.) for contouring and image analysis.

### Generation of Gross Tumor Volume in Respiratory Gating PET/CT Images

For identification of respiration-induced liver tumors, the gated PET volume was defined as the gross tumor volume (GTV). As previously mentioned, each respiratory gated PET/CT image set was sorted into 10 phases. In each phase image, GTVs were automatically contoured based on the SUV threshold. Subsequently, all delineated GTVs were verified by an in-house radiation oncologist.

### Assessment Criteria and Statistical Analysis

Two in-house experienced radiologists who are specialized in nuclear medicine were invited to perform image assessments; if there is any uncertainty, consensus between two radiologists was required. Lesions with SUV_max_ < 1.0 were considered as non-malignant lesions and discarded from downstream analysis; this threshold value was chosen based on previous studies (27, 28). The use of the SUV threshold in tumor volume segmentation eliminates intra-rater and inter-rater segmentation variability, and it is worth noting that all the generated segments in this study were approved by experienced medical oncologists with specialty in nuclear medicine. The respiratory gated and ungated images of liver lesions were analyzed and compared in terms of four parameters, namely, 1) percentage change in PET volume (Vp) and 2) percentage change on SUV_max_, SUV_mean_, and TLG values using the following equations:


(1.1) $$\%\;change\;in\;PET_{vol}=\frac{\left(ungated\;PET_{vol}-gated\;PET_{vol}\right)}{ungated\;PET_{vol}}\times 100$$

(1.2) $$\%\;change\;in\;SUV_{\max}=\frac{\left(gated\;SUV_{\max}-ungated\;SUV_{\max}\right)}{ungated\;SUV_{\max}}\times 100$$

(1.3) $$\%\;change\;in\;SUV_{mean}=\frac{\left(gated\;SUV_{mean}-ungated\;SUV_{mean}\right)}{ungated\;SUV_{mean}}\times 100$$

(1.4) $$\%\;change\;in\;TLG=\frac{\left(gated\;TLG-ungated\;TLG\right)}{ungated\;TLG}$$

### Statistical Analysis

Statistical analysis was carried out by using SPSS statistics software, version 28 (IBM Corporation, Armonk, NY, USA). Mean ± standard deviation (S.D.) of measurements was reported. Variations between respiratory gated and ungated were tested using the two-sided paired Student *t*-test. A *p* value less than 0.05 was considered as statistically significant. The correlation between three SUV parameters and TLG were evaluated by Pearson’s correlation coefficients.

## Results

### Patient Characteristic

**Figure 2:**
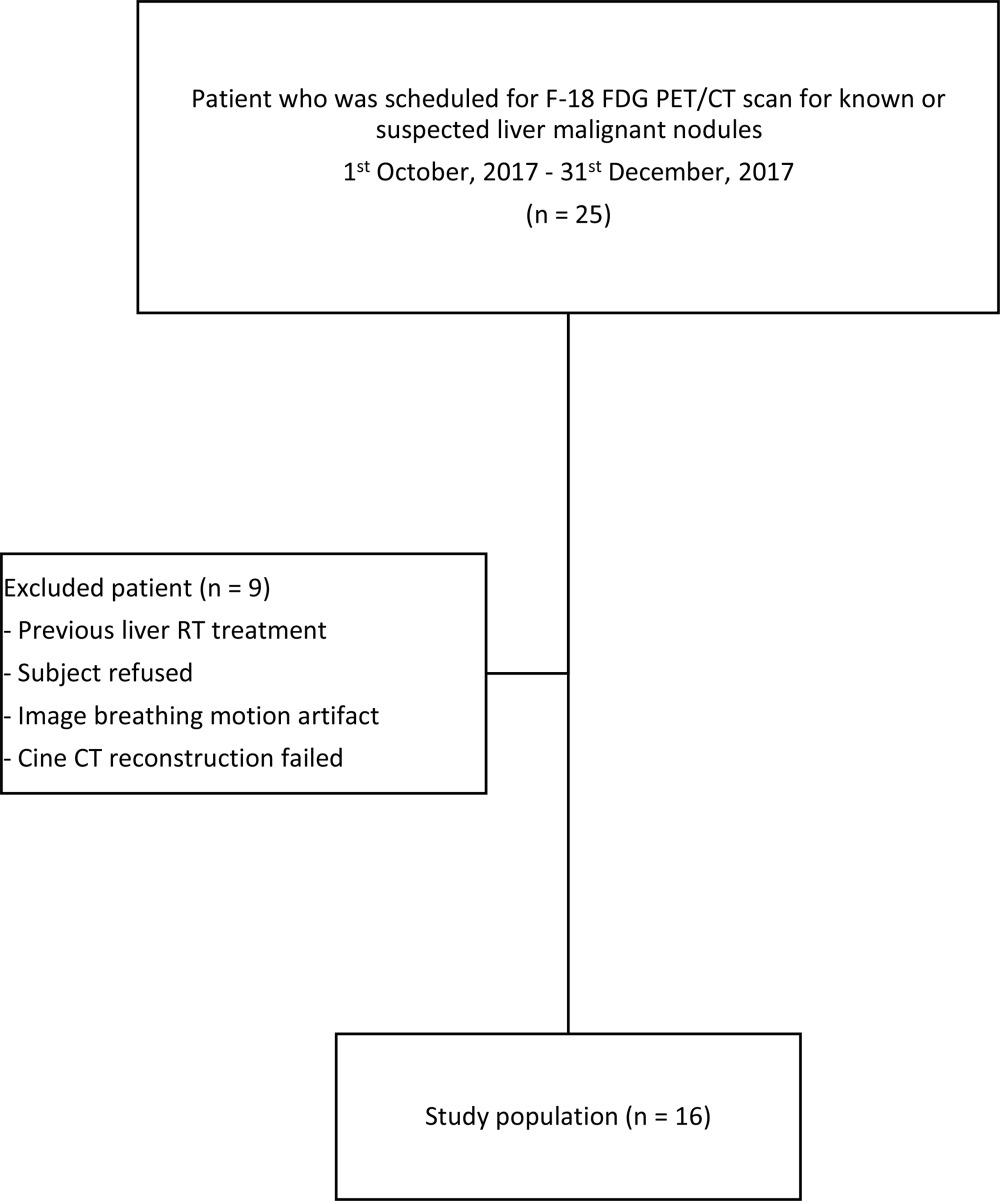
Chart on recruitment and final study population.

**Figure 3:**
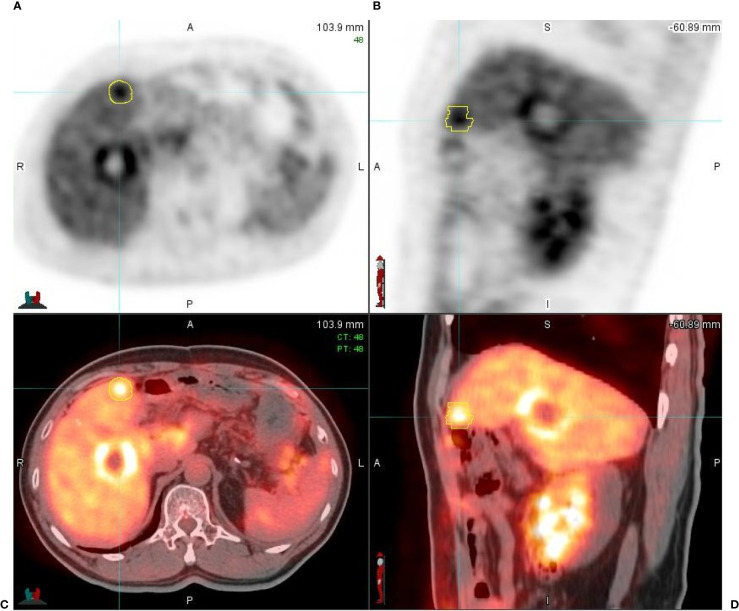
A 65-year-old male patient with hepatic metastasis from a primary rectal cancer. **(A)** Axial respiratory gated PET image (PET_0% sorted) shows two liver lesions with an increased uptake of FDG; one of the liver lesions is contoured (in yellow), measuring 2.5 cm × 2.3 cm in diameter with SUV_max_ = 9.24, conforming to liver metastasis. **(B)** Sagittal respiratory gated PET image (PET_0% sorted); one of the lesions is contoured (in yellow), measuring 2.8 cm in diameter with SUV_max_ = 9.24. **(C, D)** The respiratory gated PET axial and sagittal images fused with respiratory gated CT images (PET/CT_0% sorted); the high FDG uptake area is presented in thermal color and CT images are in gray scale.

The summary of the study population is shown in **Figure 2**. Twenty-five patients who were scheduled for receiving F-18 FDG PET/CT scan for known or suspected liver malignant diseases were recruited. Nine subjects were excluded, and the remaining study subjects (n = 16, including 9 men and 7 women) were included. The average age of the included subjects was 65 years (range: 41 to 84). The patient demographic and lesion characteristic are presented in **Table 1**. The mean total time for 4D PET/CT liver acquisition was 23 ± 2 min (range: 18.5–28.4). All successful subjects were included to perform image and uptake quantitative analyses (**Figure 3**).

**Table 1:** The patient demographic and lesion characteristics.

Patient Demographic			
Total subjects	16	Age (mean ± S.D.)	65 ± 13 (years)
Male/female	9/7	Age (range)	41–84 (years)
***Lesions characteristic***		***Primary cancer site***	
Total lesions	89	Ca rectum	4
Lesion size (mean ± S.D. *^a^*)	4.22 ± 7.46 (ml)	Ca colon	3
Lesions (range)	0.45 – 62.24 (ml)	Ca pancreas	2
**Lesion location**		Ca liver	2
Segment 1	2	Ca breast	1
Segment 2	8	Ca cecum	1
Segment 3	3	Ca lung	1
Segment 4	17	Ca sigmoid	1
Segment 5	15	Ca thyroid	1
Segment 6	21		
Segment 7	9		
Segment 8	14		

a Standard deviation (S.D.).

### Phantom Validation of the Motion Correction

From the phantom validation experiment, we observed that the SUV_max_ and SUV_mean_ values of the 4D-gating PET images were similar to the reference of static phantom images (SUV_max_: 4.97 vs. 5.13, SUV_mean_: 2.74 vs. 3.20), while those values of the ungated PET images were markedly decreased (SUV_max_: 3.97 vs. 5.13, SUV_mean_: 2.13 vs. 3.20).

### The Effect of Respiratory Gated Scan on Liver Tumors Compared to Routine 3D PET/CT Scan

The effects of respiratory gated scan on PET contour volume (Vp), SUV_max_, SUV_mean_, and total lesion glycolysis (TLG) for the 89 lesions are presented in **Table 2**. Vp (ml) was significantly decreased by using the respiratory gating technique (from 4.22 ± 7.46 to 3.32 ± 6.78, 21.48%, p < 0.001). The SUV_max_ and SUV_mean_ using the respiratory gated technique were significantly improved by 19.81% and 25.53% compared to the respiratory ungated technique. The TLG was slightly reduced from 26.37 ± 47.68 to 25.12 ± 50.59 (p = 0.1812). Details on variations of lesion volume, SUV_max_, SUV_mean_, and TLG between respiratory phases are displayed as **Supplementary Tables 1–4**, respectively, in the **Supplementary Material**.

**Table 2:** PET volume (Vp), and SUV_max_, SUV_mean_, and TLG in non-gating and gating PET/CT.

	Respiratory Ungated	Respiratory Gated	Percentage Change	p value
Total (n = 89)				
Vp (ml)	4.22 ± 7.46	3.32 ± 6.78	21.48%	<0.001
SUV_max_	8.87 ± 4.23	10.63 ± 5.25	19.81%	<0.001
SUV_mean_	5.56 ± 1.95	6.98 ± 2.94	25.53%	<0.001
TLG	26.37 ± 47.68	25.12 ± 50.59	-4.74%	0.1812
Lt lobe (n = 11)				
Vp (ml)	4.95 ± 5.69	3.88 ± 5.62	21.91%	<0.001
SUV_max_	9.15 ± 3.99	10.34 ± 4.70	12.91%	0.0049
SUV_mean_	5.45 ± 1.67	7.03 ± 2.84	29.00%	0.0034
TLG	24.67 ± 26.72	22.28 ± 30.31	-9.68%	0.1161
Rt lobe (n = 78)				
Vp (ml)	4.12 ± 7.75	3.23 ± 7.00	21.45%	<0.001
SUV_max_	8.84 ± 4.31	10.68 ± 5.38	20.82%	<0.001
SUV_mean_	5.57 ± 2.01	6.97 ± 3.00	25.06%	<0.001
TLG	26.61 ± 50.34	25.52 ± 53.27	-4.09%	0.2987

1) Values of respiratory ungated and gated were indicated as mean ± standard deviation.

2) Left (Lt) lobe (Segments 2 and 3).

3) Right (Rt) lobe (Segments 1, 4, 5, 6, 7, 8).

### Influence of Liver Tumor’s Location on the Effect of Respiratory Gating

Among the 89 studied lesions, 11 and 78 are located in the left lobe and right lobe, respectively. The average lesion size was 4.95 ± 5.69 ml for the left lobe and 4.12 ± 7.75 ml for the right lobe. The effect of respiratory gating on Vp, SUV_max_, SUV_mean_, and TLG in both lobes are presented in **Table 2**.

In the left lobe, the results showed a significant difference between ungated and gated images in Vp (4.95 ± 5.69 vs. 3.88 ± 5.62, p < 0.001), SUV_max_ (9.15 ± 3.99 vs. 10.34 ± 4.70, p = 0.0049), and SUV_mean_ (5.45 ± 1.67 vs. 7.03 ± 2.84, p = 0.0034). By contrast, ungated and gated images showed no significant difference in terms of TLG values (24.67 ± 26.72 vs. 22.28 ± 30.31, p = 0.1161).

In the right lobe, ungated and gated images in Vp showed a significant difference (4.12 ± 7.75 vs. 3.23 ± 7.00, p < 0.001), SUV_max_ (8.84 ± 4.31 vs. 10.68 ± 5.38, p < 0.001), and SUV_mean_ (5.57 ± 2.01 vs. 6.97 ± 3.00, p < 0.001). However, there was no significant difference between ungated and gated images in terms of TLG values (26.61 ± 5034 vs. 25.52 ± 53.27, p = 0.2987).

In the comparison of lesions between both sides of liver lobes, there were no significant differences in Vp (3.88 ± 5.62 vs. 3.24 ± 7.00, p = 0.7721), SUV_max_ (10.34 ± 4.70 vs. 10.68 ± 5.38, p = 0.8426), SUV_mean_ (7.03 ± 2.84 vs. 6.97 ± 2.99, p = 0.9526), and TLG (22.28 ± 30.31 vs. 25.52 ± 53.27, p = 0.8443).

### The Correlation Between % Change in SUV_max_ and 3 Quantitative Parameters on PET Image (Percentage Change in Vp, Percentage Change in SUV_mean_, and Percentage Change in TLG)

**Figure 4:**
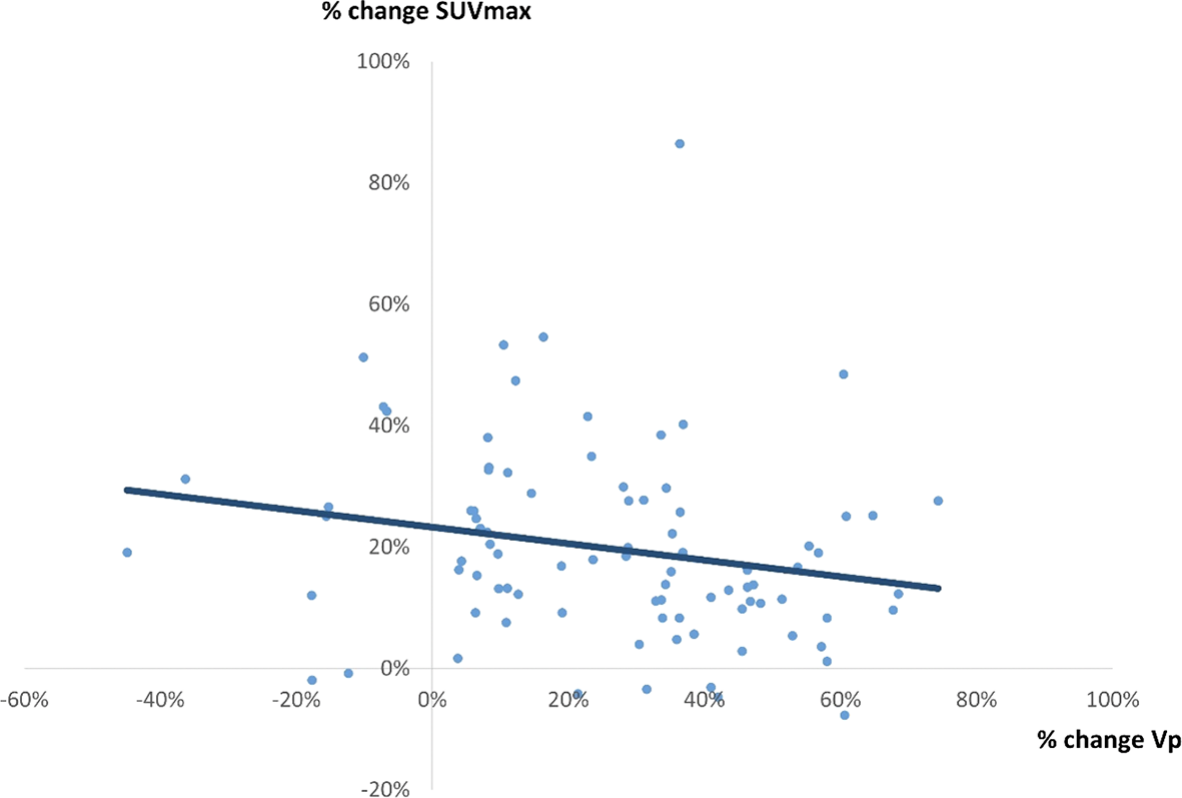
A correlation between % change in SUV_max_ and % change in Vp by 4D PET/CT scan. A weak correlation was observed (r = 0.2117, p = 0.0359, y = -0.1356x + 0.2328).

**Figure 5:**
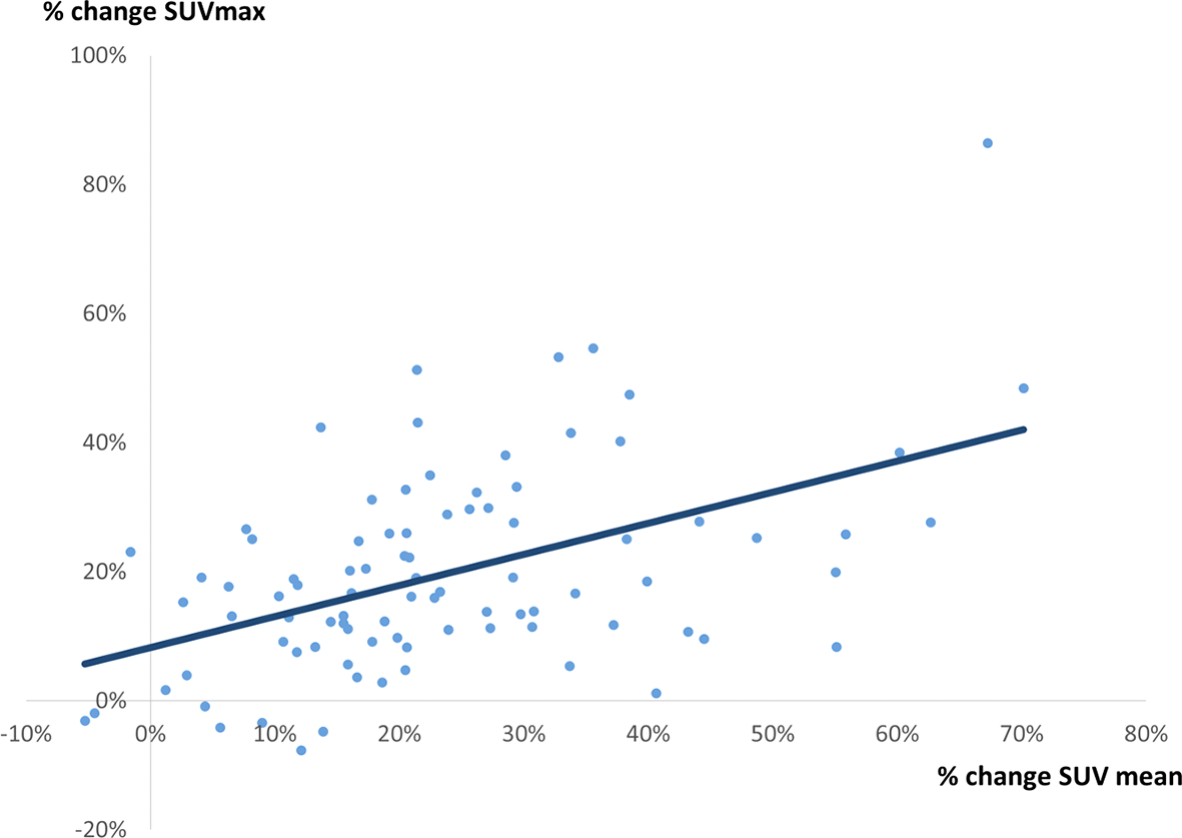
A correlation between % change in SUV_max_ and % change in SUV_mean_. A weak correlation was observed (r = 0.4891, p = 0.0897, y = 0.4815x + 0.0828).

**Figure 6:**
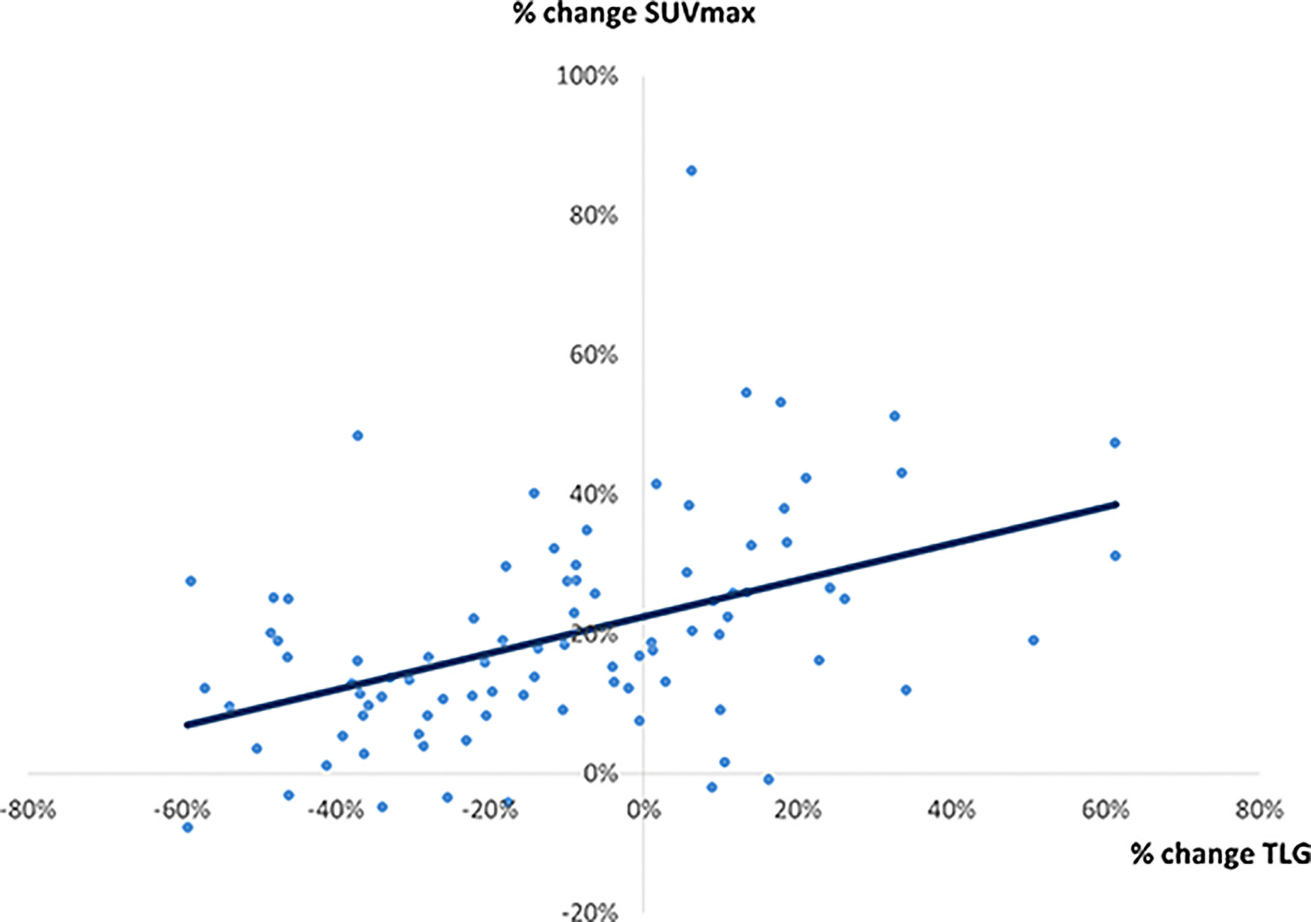
A correlation between % change in SUV_max_ and % change in TLG. A weak correlation was observed (r = 0.4522, p < 0.0001, y = 0.262x + 0.2252).

There was a weak correlation between percentage change in SUV_max_ and percentage change Vp (r = 0.2117, p = 0.0359) (**Figure 4**), between percentage change in SUV_max_ and percentage change in SUV_mean_ (r = 0.4891, p = 0.0897) (**Figure 5**), and between percentage change in SUV_max_ and percentage change in TLG (r = 0.4522, p < 0.0001) (**Figure 6**).

## Discussion

The implementation of additional gated PET/CT liver scan in routine PET/CT scan was successfully (92%) achieved. The reasons for unsuccess were gated CT image motion artifact and failure in cine CT image reconstruction. In this prospective clinical study, the gated PET/CT image demonstrated a reduction of image blurring on FDG uptake lesions. There was improvement in quantitative values including Vp, SUV_max_, and SUV_mean_. In a gated PET/CT liver study, all scans were acquired in only one bed position and the mean gated PET/CT acquisition time was about 23 min. All patients were satisfied, and duty staff had positive feedback with the workflow (29). The potential clinical applications of the gated PET images are manifold. It would allow physicians to determine an appropriate treatment regimen on a patient basis by providing better appreciation of tumor real-time trajectory in terms of both motion speed and range. Besides, the gated PET images can be used for tumor delineation with higher accuracy by providing better visualization of tumor size and border. When registered with static planning CT images, it may also allow physicians to incorporate biologic metabolism of the tumor into target delineation, paving the way toward biologic-guided RT (30).

Respiration-induced liver motion was prominent in the cranial–caudal direction. 3D PET images were acquired during multiple respiratory cycles, causing blurring of PET images, incorrect estimation of F-18 FDG uptake volume, and inaccurate lesion size determination. In the study of a total of 89 lesions, the gated PET illustrated a significant reduction in PET volume by 21.48% compared to the ungated PET. Furthermore, the gated PET images were sharper, with a more well-defined tumor boarder. Our findings are in line with previous 4D PET/CT studies which reported a mean reduction of 11%–45% in the gated PET volume compared to the non-gated PET volume (31–33). In addition, radiological information from PET/CT images is commonly utilized in radiotherapy treatment planning, especially when radiation oncologists delineate the gross target volume (GTV) and clinical target volume (CTV) and estimate tumor motion during respiration (34). The improvement of visual diagnostic value in the gated PET/CT was also in agreement with the finding reported by Fin et al., although PET quantitative analysis was not described (35). Therefore, we believe that the gated PET images could be a routine scan when liver radiotherapy treatment is intended, especially when the treatment was determined to be respiratory-gated (36, 37). Although additional time for patient setup and scan is required, respiratory gated scan can be implemented feasibly and efficiently in clinical routines. Gated PET/CT also has a great potential for giving higher spatial resolution and motion artifact free images, which is an essence of future development of molecular imaging.

It is worth mentioning that recent advances in PET cameras and AI techniques have enabled a data-driven (i.e., device-less) approach, such as MotionFree™/data-driven gating (DDG), for respiratory gating in PET images, potentially serving as an alternative to the classical device-based gating system, such as the Varian RPM-based approach. Both approaches have been studied for gated PET imaging. For instance, Crivellaro et al. retrospectively analyzed standard 3D-PET/CT (i.e., ungated) and liver 4D-PET/CT (i.e., gated) images of 56 patients, hoping to investigate the added diagnostic value of device-based respiratory-gated 4D PET/CT in detecting and characterizing a total of 72 liver lesions (21). They reported that an enhanced confidence of physicians in lesion detection on the gated PET/CT was found in 51.4% of the studied lesions, compared to the ungated PET/CT. Besides, they also demonstrated a significantly higher level of the SUV_max_ value for liver lesions in the gated PET, therefore improving quantitative characterization of the lesions, in comparison to the ungated PET (21). In addition, Michael et al. conducted a retrospective analysis on 149 cancer patients to evaluate the impact of device-less respiratory gating (DDG) on PET image quality and lesion detection (22). They reported that the issue of image blurring in PET images was significantly lower when DDG (i.e., gated) was used, compared to the PET images without DDG application (i.e., ungated). Besides, boundary of organs, including liver and spleen, was rated significantly sharper on the DDG-gated PET images than those on the ungated PET images (22).

Both device-based and device-less approaches have their advantages and drawbacks. Unlike the Varian RPM-based (i.e., device-based) respiratory gating technique, the device-less approach waives the requirement of setting up the respiratory gating device on the patient’s body which prolongs the scanning time, making this approach appear to be more patient-friendly. Nevertheless, the device-based system has long been considered as a standard procedure for correcting respiratory motion-induced uncertainties in PET images in clinics at present, and hence, it is a more popular and widely used technique, compared to the device-less gating technique. Furthermore, the device-based system possesses a unique capability in achieving breathing-synchronized RT treatment (38), while the device-less system is not used in RT application. This feature is of high clinical value in realizing real-time tumor trajectory, and the treating oncologist can therefore be more confident in delineating the internal target volume (ITV) of the tumor, which would eventually translate to the patient’s treatment outcome benefit. Besides, the information of real-time tumor trajectory also allows physicians to better appreciate the speed of the tumor motion, based on which the physicians can better determine an appropriate treatment regimen (e.g., whether to treat with SBRT or not) on a case-by-case basis. All things considered, although the device-less approach appears to be more patient-friendly, it is not presently used for radiotherapy purposes. In the context of radiotherapy, other aspects, such as reliability and treatment efficacy, are more important, and thus we believe that the Varian RPM-based respiratory gating system still plays a key role in managing liver malignancies.

Previously, gated PET/CT images were found to produce a more accurate evaluation of FDG uptake in liver tumors that may allow physicians to make better tumor characterization, more personalized treatment strategy, treatment response monitoring, and prediction of survival (9, 31, 39). In this study, respiratory gating improved PET image measurement of tumor SUV and metabolic volume. The SUV value is a sensitive indicator to represent tumor metabolism or even tumor proliferation. In our gated PET image analysis, the mean value of SUV_max_ was increased by 19.81% compared to the ungated PET image. This finding is in line with that reported by Suenaga et al., in which a 22% increase in SUV_max_ value was reported (34). However, the SUV_max_ value represents the local maximum of a region of interest, which means that it might only reflect a single voxel value of maximum FDG uptake within the entire tumor (40, 41). By contrast, SUV_mean_ is another indictor to represent the tumor’s metabolic activity as a whole. In the present study, respiratory gating improved the mean value of SUV_mean_ by 25.53%. The deviation of FDG uptake results through respiratory gating between our finding and previous studies could be related to the following two reasons. First, the FDG uptake in liver tumor may change with PET scan time; a delayed gated PET acquisition protocol could result in a higher SUV value (42, 43). In this study, all gated PET/CT acquisition was performed after routine ungated whole-body FDG scan. Second, as suggested by Guerra et al., differences in tumor type, volume, geographical region, elasticity, and motion could influence the percentage change of the respiratory gated results (42).

In previous reports, the prognostic value of TLG on preoperative F-18 FDG PET/CT has been widely studied for estimating intrahepatic recurrence-free survival (IHRFS), extrahepatic metastasis-free survival (EHMFS), and overall survival (OS) in patients with liver malignancies (44–46). Therefore, it is important for physicians to identify TLG and compare clinical findings with other pathological or histological prognostic factors. In this study, we evaluated the percentage change in TLG when the 4D PET/CT technique was introduced. The mean of the TLG value decreased by 4.74% in a total of 89 lesions, although no significant difference was observed. The decrease in the tumor’s TLG values could probably be due to the reduction in PET volume in the respiratory gated scan.

On the other hand, the correlation with percentage change in SUV_max_ was stronger in percentage change in SUV_mean_ (r = 0.49) and TLG (r = 0.45) than percentage change in Vp (r = 0.21) in this study. These findings indicated that the 4D PET/CT scan may provide a generally higher SUV value, which has a stronger effect on the lesion’s uptake improvement than the effect on the reduction of PET volume. Apart from this, we have assessed the correlation between amplitude of motion and SUV differences between gated and ungated images (**Supplementary Figures 1, 2**). It was observed that the correlation was not strong (R = 0.4543 for SUV_mean_ and 0.5016 for SUV_max_). We speculated that this finding could be probably attributed to the intrinsic property of PET images. Unlike CT images where imaging voxels are characterized by a well-defined absolute value of Hounsfield unit, PET images commonly suffer from high imaging noise and is susceptible to injection dose of the radioactive agents, leading to a highly unstable imaging voxel intensity. In view of this, image normalization between patients and hence comparison of SUV values between patients are practically challenging. Therefore, it would be difficult to obtain a high correlation between the SUV differences and the motion amplitude of lesion. Although we found that the correlation was 0.4543 for SUV_mean_ and 0.5015 for SUV_max_, we think that these values are still considered reasonable due to the intrinsic unstable property of PET images.

There were several limitations in the present study. First, the patient population was relatively small, although a total of 89 lesions were studied. Future evaluation of a large cohort is recommended to evaluate the feasibility and clinical benefits of respiratory gated PET/CT for detection of liver malignancies. Second, although the digital phantom was adopted for validation in this study because of its wide-spreading application (47, 48), further investigation on a 4D eXtended CArdiac-Torso (XCAT) phantom is warranted to yield a more validated result. Third, the choice of the SUV threshold value for lesion contouring might affect the quantitative measurements in this study, although all the final contours were verified by a nuclear medicine physician. Lastly, this work remains to be a feasibility study in nature; optimal settings of reconstruction parameters and function for PET gating were not investigated in this study. Further explorations in this regard are highly encouraged to strengthen the clinical value of the gated PET in the future.

In conclusion, we have demonstrated the feasibility of implementing 4D PET/CT scan for liver malignancies in a prospective clinical study. The 4D PET/CT was found to mitigate issues of image blurring artifact and improve the accuracy of lesion volume on PET images. The improved accuracy in the classification and identification of liver tumors with 4D PET image would potentially lead to its increased utilization in target delineation of GTV, ITV, and PTV for liver radiotherapy treatment planning in the future.

## Data Availability Statement

The original contributions presented in the study are included in the article/**Supplementary Material**. Further inquiries can be directed to the corresponding author.

## Ethics Statement

The studies involving human participants were reviewed and approved by 1) the Hong Kong Polytechnic University - human subjects ethics sub-committee and 2) Hong Kong Baptist Hospital - Clinical and research ethics committee. The patients/participants provided their written informed consent to participate in this study.

## Author Contributions

JC was the chief supervisor in the study. AHC and VW contributed to the conception and methodology of the study. AHC and VW applied the ethical approval. AHC organized the patient recruitment and data collection process. AHC and ALC performed the data analysis. AHC wrote the first draft of the manuscript. ALC revised the content. JC revised and approved the submitted version. All authors contributed to the article and approved the submitted version.

## Conflict of Interest

The authors declare that the research was conducted in the absence of any commercial or financial relationships that could be construed as a potential conflict of interest.

## Publisher’s Note

All claims expressed in this article are solely those of the authors and do not necessarily represent those of their affiliated organizations, or those of the publisher, the editors and the reviewers. Any product that may be evaluated in this article, or claim that may be made by its manufacturer, is not guaranteed or endorsed by the publisher.
