# Supplementary material for: Respiratory 4D-Gating F-18 FDG PET/CT Scan for Liver Malignancies: Feasibility in Liver Cancer Patient and Tumor Quantitative Analysis
Source: Front Oncol. 2022 Feb 9;12:789506. doi: 10.3389/fonc.2022.789506 (PMC8864173; doi:10.3389/fonc.2022.789506)
Supplement: Supplementary file 1 [file DataSheet_1.docx]

**Supplementary Material for “Respiratory 4D-gating F-18 FDG PET/CT scan for liver malignancies: Feasibility in liver cancer patient and tumor quantitative analysis”**

**Supplementary Table 1:**

**Supplementary Table 1.** Ungated lesion volume, variations of lesion volumes between respiratory phases, and averaged lesion volume averaged over the 10 phases for all the 89 lesions in this study.

| **Subject (Sub)** | ***lesion*** | **Ungated volume (ml)** | **Gated volume (ml) across respiratory phases** | | | | | | | | | | **Averaged gated volume over the 10 phases** |
| --- | --- | --- | --- | --- | --- | --- | --- | --- | --- | --- | --- | --- | --- |
|  |  |  | **10%** | **20%** | **30%** | **40%** | **50%** | **60%** | **70%** | **80%** | **90%** | **100%** |  |
| Sub-1 | *1* | 2.80 | 1.94 | 1.90 | 1.97 | 2.01 | 2.12 | 2.21 | 2.13 | 1.91 | 1.98 | 1.97 | 2.01 |
|  | *2* | 4.30 | 4.05 | 3.42 | 3.93 | 3.92 | 3.70 | 3.58 | 3.89 | 3.54 | 4.10 | 4.13 | 3.83 |
|  | *3* | 0.97 | 0.44 | 0.65 | 0.84 | 0.61 | 0.77 | 0.59 | 0.81 | 0.78 | 0.66 | 0.60 | 0.68 |
|  | *4* | 2.20 | 2.02 | 1.51 | 2.06 | 2.05 | 1.92 | 1.95 | 2.05 | 2.16 | 2.21 | 2.63 | 2.06 |
|  | *5* | 0.65 | 0.69 | 0.53 | 0.45 | 0.55 | 0.57 | 0.59 | 0.55 | 0.53 | 0.60 | 0.62 | 0.57 |
| Sub-2 | *1* | 0.80 | 0.84 | 0.62 | 0.74 | 0.70 | 0.78 | 0.68 | 0.65 | 0.68 | 0.65 | 0.68 | 0.70 |
|  | *2* | 2.19 | 1.69 | 1.48 | 1.41 | 1.29 | 1.37 | 1.42 | 1.38 | 1.24 | 1.27 | 1.38 | 1.39 |
|  | *3* | 2.44 | 1.35 | 1.19 | 1.88 | 1.98 | 1.96 | 2.25 | 2.14 | 1.89 | 1.86 | 2.33 | 1.88 |
|  | *4* | 5.98 | 5.25 | 5.61 | 5.79 | 5.66 | 6.19 | 5.35 | 6.38 | 5.46 | 5.09 | 5.63 | 5.64 |
|  | *5* | 1.61 | 1.42 | 1.54 | 1.63 | 1.47 | 1.60 | 1.37 | 1.37 | 1.48 | 1.43 | 1.45 | 1.48 |
|  | *6* | 1.40 | 0.92 | 0.91 | 0.96 | 0.95 | 0.88 | 0.93 | 0.89 | 0.92 | 0.88 | 0.82 | 0.91 |
|  | *7* | 3.47 | 3.82 | 3.21 | 3.29 | 3.01 | 3.20 | 3.07 | 3.09 | 3.06 | 2.92 | 3.16 | 3.18 |
|  | *8* | 5.14 | 3.58 | 3.66 | 3.66 | 3.94 | 3.67 | 4.17 | 4.24 | 3.88 | 4.30 | 4.17 | 3.93 |
|  | *9* | 1.93 | 1.86 | 1.89 | 1.82 | 1.91 | 1.61 | 1.69 | 1.83 | 1.65 | 1.79 | 1.70 | 1.78 |
|  | *10* | 2.77 | 3.01 | 2.57 | 2.59 | 2.48 | 2.45 | 2.55 | 2.60 | 2.51 | 2.79 | 2.45 | 2.60 |
|  | *11* | 2.33 | 2.21 | 2.29 | 2.22 | 2.17 | 2.13 | 1.90 | 2.08 | 2.11 | 2.01 | 2.20 | 2.13 |
|  | *12* | 0.85 | 0.79 | 0.76 | 0.78 | 0.88 | 0.86 | 0.67 | 0.66 | 0.57 | 0.67 | 0.62 | 0.73 |
| Sub-3 | *1* | 17.47 | 18.63 | 20.54 | 18.88 | 20.77 | 19.47 | 20.83 | 19.95 | 20.29 | 21.48 | 20.52 | 20.14 |
|  | *2* | 19.73 | 19.97 | 19.26 | 14.92 | 19.40 | 17.64 | 17.93 | 18.89 | 19.17 | 18.56 | 18.89 | 18.46 |
| Sub-4 | *1* | 3.65 | 2.64 | 4.57 | 4.22 | 5.50 | 2.65 | 4.30 | 4.06 | 4.62 | 4.32 | 3.32 | 4.02 |
|  | *2* | 1.25 | 1.16 | 1.14 | 1.11 | 1.15 | 1.22 | 1.25 | 1.04 | 1.00 | 1.00 | 1.12 | 1.12 |
|  | *3* | 0.56 | 0.40 | 0.78 | 0.43 | 0.49 | 0.35 | 0.41 | 0.33 | 0.35 | 0.35 | 0.40 | 0.43 |
|  | *4* | 2.98 | 1.64 | 1.56 | 1.70 | 1.65 | 2.10 | 2.25 | 2.07 | 1.96 | 2.33 | 1.57 | 1.88 |
|  | *5* | 2.55 | 3.25 | 3.01 | 2.60 | 2.87 | 2.85 | 2.90 | 2.99 | 2.93 | 2.91 | 3.17 | 2.95 |
|  | *6* | 0.97 | 0.64 | 0.40 | 0.76 | 1.08 | 1.36 | 1.48 | 1.35 | 1.08 | 1.20 | 1.05 | 1.04 |
|  | *7* | 1.01 | 0.76 | 0.94 | 0.61 | 0.78 | 0.81 | 0.79 | 0.99 | 0.74 | 0.82 | 0.70 | 0.79 |
|  | *8* | 1.51 | 1.29 | 1.33 | 1.44 | 1.47 | 1.42 | 1.46 | 1.35 | 1.40 | 1.39 | 1.32 | 1.39 |
|  | *9* | 1.26 | 1.29 | 1.70 | 1.88 | 1.81 | 1.72 | 1.73 | 1.82 | 1.72 | 1.80 | 1.70 | 1.72 |
|  | *10* | 1.33 | 1.61 | 1.33 | 1.33 | 1.41 | 1.55 | 1.52 | 1.27 | 1.50 | 1.36 | 1.31 | 1.42 |
|  | *11* | 2.86 | 2.31 | 2.58 | 2.17 | 2.57 | 3.00 | 2.97 | 2.73 | 3.00 | 2.51 | 2.00 | 2.58 |
|  | *12* | 0.45 | 0.35 | 0.37 | 0.38 | 0.39 | 0.30 | 0.31 | 0.33 | 0.35 | 0.36 | 0.35 | 0.35 |
|  | *13* | 1.30 | 1.64 | 1.56 | 1.70 | 1.65 | 2.10 | 2.25 | 2.07 | 1.96 | 2.33 | 1.57 | 1.88 |
| Sub-5 | *1* | 4.07 | 2.65 | 3.04 | 2.64 | 1.73 | 1.55 | 1.68 | 1.71 | 2.32 | 2.28 | 2.56 | 2.22 |
|  | *2* | 2.61 | 1.52 | 1.81 | 1.64 | 2.17 | 1.35 | 1.51 | 1.47 | 1.62 | 1.76 | 1.87 | 1.67 |
| Sub-6 | *1* | 2.38 | 1.29 | 1.66 | 1.42 | 1.55 | 1.54 | 1.64 | 1.73 | 1.78 | 1.62 | 1.76 | 1.60 |
| Sub-7 | *1* | 2.21 | 1.66 | 1.85 | 2.09 | 1.62 | 2.25 | 1.78 | 1.80 | 1.69 | 1.58 | 1.58 | 1.79 |
| Sub-8 | *1* | 2.27 | 0.96 | 1.37 | 0.94 | 1.04 | 0.90 | 0.84 | 1.04 | 1.23 | 1.28 | 0.91 | 1.05 |
|  | *2* | 1.69 | 1.12 | 0.88 | 0.83 | 0.54 | 1.13 | 1.01 | 1.03 | 0.85 | 1.21 | 0.94 | 0.95 |
|  | *3* | 3.40 | 1.93 | 1.75 | 2.00 | 2.17 | 1.85 | 1.75 | 1.77 | 1.82 | 1.67 | 1.80 | 1.85 |
|  | *4* | 4.17 | 1.59 | 1.48 | 1.48 | 1.88 | 1.61 | 2.00 | 2.19 | 1.76 | 1.88 | 1.99 | 1.79 |
|  | *5* | 2.80 | 1.91 | 1.23 | 1.27 | 1.80 | 1.92 | 2.03 | 2.09 | 1.48 | 2.10 | 1.40 | 1.72 |
|  | *6* | 0.47 | 1.44 | 0.81 | 0.34 | 0.24 | 0.21 | 0.25 | 0.84 | 0.44 | 0.52 | 0.44 | 0.55 |
|  | *7* | 3.41 | 2.39 | 2.64 | 1.96 | 1.91 | 1.93 | 2.24 | 1.90 | 2.04 | 2.19 | 2.51 | 2.17 |
|  | *8* | 10.10 | 9.01 | 9.29 | 8.76 | 7.70 | 9.23 | 9.74 | 9.28 | 9.16 | 8.75 | 9.11 | 9.00 |
|  | *9* | 1.70 | 1.52 | 1.29 | 1.40 | 1.37 | 1.20 | 1.23 | 1.07 | 1.42 | 1.64 | 1.61 | 1.38 |
| Sub-9 | *1* | 4.00 | 3.99 | 3.84 | 4.18 | 3.87 | 4.45 | 3.92 | 3.07 | 3.08 | 4.50 | 3.60 | 3.85 |
| Sub-10 | *1* | 7.47 | 6.54 | 7.81 | 8.06 | 7.90 | 6.19 | 6.25 | 6.69 | 5.01 | 7.34 | 7.64 | 6.94 |
|  | *2* | 4.07 | 3.88 | 3.67 | 3.78 | 3.56 | 3.86 | 3.75 | 3.92 | 4.03 | 3.64 | 3.93 | 3.80 |
|  | *3* | 1.95 | 1.85 | 1.89 | 1.89 | 1.83 | 1.93 | 1.62 | 2.02 | 1.79 | 2.28 | 1.56 | 1.87 |
| Sub-11 | *1* | 9.31 | 8.02 | 8.39 | 6.78 | 7.71 | 8.20 | 7.68 | 6.99 | 7.70 | 8.06 | 8.35 | 7.79 |
|  | *2* | 2.95 | 2.07 | 1.62 | 2.02 | 2.10 | 1.89 | 2.09 | 1.77 | 2.40 | 1.78 | 1.61 | 1.94 |
|  | *3* | 1.79 | 1.60 | 0.92 | 0.66 | 0.72 | 0.68 | 1.58 | 1.46 | 0.61 | 0.68 | 0.70 | 0.96 |
|  | *4* | 3.69 | 1.32 | 1.41 | 1.01 | 1.39 | 1.47 | 1.15 | 1.82 | 1.63 | 1.79 | 1.46 | 1.45 |
|  | *5* | 2.85 | 1.81 | 1.28 | 1.95 | 1.65 | 1.37 | 1.69 | 1.52 | 1.41 | 1.37 | 1.12 | 1.52 |
|  | *6* | 11.55 | 6.04 | 5.63 | 6.22 | 6.10 | 7.08 | 6.38 | 6.74 | 5.73 | 5.78 | 6.29 | 6.20 |
|  | *7* | 5.01 | 2.33 | 2.07 | 2.64 | 1.88 | 1.94 | 1.93 | 2.32 | 2.60 | 2.67 | 1.97 | 2.24 |
|  | *8* | 2.62 | 1.33 | 1.62 | 1.62 | 1.91 | 1.54 | 2.17 | 1.62 | 2.24 | 2.14 | 1.76 | 1.80 |
| Sub-12 | *1* | 2.15 | 1.45 | 0.86 | 1.78 | 1.09 | 1.57 | 0.97 | 1.78 | 1.09 | 1.06 | 1.05 | 1.27 |
| Sub-13 | *1* | 0.86 | 1.01 | 1.14 | 1.18 | 0.76 | 0.79 | 1.25 | 0.89 | 1.00 | 0.87 | 0.77 | 0.97 |
|  | *2* | 1.63 | 0.58 | 0.88 | 0.77 | 0.56 | 0.59 | 0.55 | 0.59 | 0.90 | 0.49 | 0.93 | 0.68 |
|  | *3* | 2.21 | 0.96 | 1.46 | 1.42 | 0.78 | 1.17 | 0.80 | 0.75 | 1.08 | 0.84 | 1.14 | 1.04 |
|  | *4* | 13.56 | 8.93 | 9.01 | 9.05 | 8.75 | 8.91 | 8.81 | 9.01 | 9.34 | 8.97 | 9.15 | 8.99 |
|  | *5* | 7.75 | 4.08 | 4.04 | 4.00 | 4.24 | 4.43 | 4.16 | 4.15 | 3.87 | 4.12 | 3.85 | 4.09 |
|  | *6* | 2.38 | 1.62 | 1.19 | 1.23 | 1.35 | 1.42 | 1.23 | 1.14 | 1.58 | 1.89 | 1.40 | 1.41 |
|  | *7* | 3.37 | 1.12 | 1.95 | 1.38 | 1.39 | 1.26 | 1.10 | 1.44 | 1.61 | 1.48 | 1.84 | 1.46 |
|  | *8* | 2.24 | 0.88 | 0.85 | 0.85 | 0.85 | 0.93 | 0.68 | 0.51 | 0.70 | 0.98 | 0.66 | 0.79 |
|  | *9* | 0.84 | 0.31 | 0.55 | 0.57 | 0.41 | 0.44 | 0.58 | 0.46 | 0.47 | 0.62 | 0.46 | 0.49 |
|  | *10* | 2.84 | 1.51 | 1.35 | 1.62 | 1.25 | 1.63 | 1.38 | 1.57 | 1.30 | 1.50 | 1.59 | 1.47 |
|  | *11* | 1.16 | 0.36 | 0.65 | 0.50 | 0.42 | 0.71 | 0.34 | 0.37 | 0.37 | 0.61 | 0.24 | 0.46 |
|  | *12* | 4.15 | 1.84 | 2.16 | 2.28 | 2.58 | 2.22 | 2.11 | 2.15 | 2.32 | 2.43 | 2.24 | 2.23 |
|  | *13* | 3.13 | 1.58 | 1.69 | 1.87 | 1.42 | 1.37 | 1.59 | 1.36 | 1.41 | 1.35 | 1.57 | 1.52 |
| Sub-14 | *1* | 1.01 | 0.33 | 0.23 | 0.24 | 0.19 | 0.23 | 0.20 | 0.25 | 0.41 | 0.30 | 0.21 | 0.26 |
|  | *2* | 0.84 | 0.36 | 0.26 | 0.29 | 0.21 | 0.26 | 0.23 | 0.30 | 0.27 | 0.30 | 0.23 | 0.27 |
|  | *3* | 2.16 | 1.99 | 1.00 | 1.34 | 0.93 | 1.42 | 1.36 | 0.91 | 1.53 | 1.19 | 1.97 | 1.36 |
|  | *4* | 18.06 | 20.31 | 20.76 | 20.23 | 20.17 | 19.84 | 20.04 | 19.76 | 20.69 | 20.17 | 19.83 | 20.18 |
|  | *5* | 0.84 | 0.39 | 1.11 | 0.40 | 0.88 | 0.21 | 0.18 | 0.39 | 0.95 | 0.84 | 0.44 | 0.58 |
|  | *6* | 0.84 | 1.08 | 1.13 | 0.42 | 1.14 | 1.04 | 0.98 | 1.17 | 1.02 | 0.90 | 1.01 | 0.99 |
|  | *7* | 19.76 | 11.28 | 12.90 | 13.32 | 12.87 | 13.01 | 11.85 | 12.13 | 12.61 | 12.46 | 13.21 | 12.56 |
|  | *8* | 6.21 | 3.94 | 4.26 | 4.23 | 4.34 | 3.23 | 4.13 | 4.16 | 4.33 | 4.38 | 4.21 | 4.12 |
|  | *9* | 1.16 | 0.26 | 0.41 | 0.25 | 0.18 | 0.57 | 0.76 | 0.24 | 0.27 | 0.85 | 0.80 | 0.46 |
|  | *10* | 1.00 | 0.31 | 1.28 | 0.31 | 0.33 | 0.34 | 0.27 | 0.81 | 1.10 | 1.28 | 1.12 | 0.72 |
|  | *11* | 1.59 | 1.22 | 1.16 | 1.02 | 1.05 | 1.19 | 0.93 | 1.19 | 1.17 | 1.19 | 1.21 | 1.13 |
|  | *12* | 1.89 | 1.23 | 1.38 | 1.28 | 1.15 | 1.21 | 1.24 | 1.22 | 1.31 | 1.31 | 1.18 | 1.25 |
|  | *13* | 2.23 | 1.04 | 0.99 | 0.87 | 1.04 | 0.89 | 0.79 | 0.92 | 0.87 | 1.01 | 0.94 | 0.94 |
| Sub-15 | *1* | 62.24 | 57.48 | 55.91 | 57.73 | 55.33 | 53.38 | 55.17 | 57.43 | 56.40 | 56.05 | 56.84 | 56.17 |
| Sub-16 | *1* | 1.98 | 1.50 | 1.45 | 1.59 | 1.20 | 2.14 | 0.97 | 1.60 | 1.53 | 0.97 | 1.13 | 1.41 |
|  | *2* | 0.79 | 0.44 | 0.26 | 0.18 | 0.19 | 0.38 | 0.26 | 0.22 | 0.18 | 0.19 | 0.19 | 0.25 |
|  | *3* | 1.41 | 1.15 | 1.13 | 0.97 | 0.98 | 0.78 | 0.78 | 0.78 | 0.77 | 0.98 | 0.95 | 0.93 |
|  | *4* | 1.41 | 1.16 | 0.90 | 0.80 | 1.02 | 0.88 | 0.87 | 0.70 | 0.88 | 0.88 | 1.06 | 0.92 |

**Supplementary Table 2:**

**Supplementary Table 2.** Ungated SUVmax, variations of SUVmax between respiratory phases, and averaged SUVmax averaged over the 10 phases for all the 89 lesions in this study.

| **Subject (Sub)** | ***lesion*** | **Ungated SUVmax** | **SUVmax across respiratory phases** | | | | | | | | | | | **Averaged SUVmax over the 10 phases** |
| --- | --- | --- | --- | --- | --- | --- | --- | --- | --- | --- | --- | --- | --- | --- |
|  |  |  | **10%** | **20%** | **30%** | **40%** | **50%** | **60%** | **70%** | **80%** | **90%** | **100%** |  | |
| Sub-1 | *1* | 19.93 | 29.89 | 26.89 | 26.82 | 25.14 | 23.00 | 21.91 | 27.13 | 27.00 | 26.82 | 24.28 | 25.89 | |
|  | *2* | 8.90 | 11.09 | 10.44 | 9.21 | 10.04 | 10.25 | 9.49 | 9.65 | 10.59 | 9.75 | 10.22 | 10.07 | |
|  | *3* | 8.77 | 11.85 | 9.45 | 8.76 | 9.04 | 8.37 | 8.04 | 8.86 | 8.79 | 8.24 | 9.77 | 9.12 | |
|  | *4* | 8.08 | 9.82 | 9.44 | 8.42 | 7.84 | 8.98 | 8.72 | 8.06 | 9.53 | 8.73 | 9.65 | 8.92 | |
|  | *5* | 7.88 | 9.95 | 9.61 | 9.52 | 7.45 | 8.90 | 6.97 | 8.36 | 9.23 | 9.41 | 9.05 | 8.85 | |
| Sub-2 | *1* | 6.48 | 8.70 | 8.70 | 8.64 | 9.05 | 10.48 | 9.89 | 7.88 | 10.28 | 11.14 | 10.77 | 9.55 | |
|  | *2* | 7.18 | 11.41 | 11.97 | 13.77 | 14.72 | 14.39 | 14.84 | 11.69 | 14.41 | 13.23 | 13.44 | 13.39 | |
|  | *3* | 8.86 | 10.90 | 16.08 | 12.83 | 12.13 | 11.07 | 11.64 | 11.44 | 15.13 | 11.98 | 12.20 | 12.54 | |
|  | *4* | 7.39 | 7.99 | 9.32 | 9.79 | 10.65 | 8.25 | 9.67 | 7.76 | 11.08 | 10.41 | 8.19 | 9.31 | |
|  | *5* | 6.20 | 9.91 | 6.79 | 7.82 | 7.73 | 7.47 | 8.73 | 7.41 | 10.02 | 8.11 | 8.57 | 8.26 | |
|  | *6* | 4.75 | 6.79 | 5.41 | 5.52 | 6.76 | 5.11 | 5.67 | 5.76 | 5.95 | 5.80 | 5.28 | 5.81 | |
|  | *7* | 8.73 | 12.25 | 10.44 | 11.86 | 12.56 | 10.77 | 12.62 | 11.87 | 13.08 | 10.72 | 9.67 | 11.58 | |
|  | *8* | 10.99 | 13.58 | 13.52 | 13.80 | 6.54 | 12.44 | 12.59 | 14.67 | 15.68 | 12.94 | 13.87 | 12.96 | |
|  | *9* | 6.37 | 8.55 | 7.26 | 8.09 | 7.59 | 7.74 | 6.98 | 7.22 | 9.08 | 7.53 | 7.98 | 7.80 | |
|  | *10* | 10.22 | 12.17 | 14.62 | 12.65 | 13.19 | 12.00 | 12.14 | 11.54 | 16.03 | 12.55 | 11.81 | 12.87 | |
|  | *11* | 9.09 | 11.40 | 11.83 | 11.07 | 11.16 | 10.07 | 10.51 | 10.22 | 9.04 | 10.58 | 13.65 | 10.95 | |
|  | *12* | 5.27 | 5.67 | 5.78 | 5.90 | 6.80 | 6.87 | 6.47 | 6.31 | 9.20 | 8.26 | 6.65 | 6.79 | |
| Sub-3 | *1* | 7.59 | 9.34 | 8.64 | 9.73 | 10.57 | 8.81 | 9.36 | 11.14 | 10.12 | 8.82 | 9.57 | 9.61 | |
|  | *2* | 7.41 | 8.81 | 8.67 | 9.73 | 9.26 | 9.51 | 8.77 | 9.60 | 9.93 | 8.83 | 9.30 | 9.24 | |
| Sub-4 | *1* | 6.07 | 8.65 | 8.21 | 8.39 | 9.00 | 8.70 | 8.01 | 11.12 | 10.98 | 8.82 | 9.93 | 9.18 | |
|  | *2* | 7.89 | 10.89 | 14.25 | 10.67 | 9.95 | 11.10 | 12.66 | 12.20 | 16.53 | 9.61 | 13.07 | 12.09 | |
|  | *3* | 5.45 | 6.33 | 5.83 | 8.02 | 7.70 | 7.70 | 7.53 | 6.16 | 7.80 | 9.16 | 7.31 | 7.35 | |
|  | *4* | 7.86 | 8.69 | 10.30 | 8.49 | 8.85 | 7.70 | 10.46 | 9.92 | 11.76 | 9.16 | 8.29 | 9.36 | |
|  | *5* | 14.33 | 14.60 | 15.64 | 17.76 | 20.21 | 15.39 | 20.69 | 19.26 | 18.47 | 16.97 | 20.23 | 17.92 | |
|  | *6* | 5.56 | 5.49 | 4.73 | 8.06 | 5.58 | 8.67 | 8.21 | 10.78 | 12.46 | 9.43 | 6.17 | 7.96 | |
|  | *7* | 5.98 | 5.63 | 4.92 | 6.74 | 5.29 | 5.65 | 5.32 | 4.87 | 6.74 | 6.32 | 5.83 | 5.73 | |
|  | *8* | 7.17 | 8.58 | 9.27 | 8.02 | 10.50 | 10.53 | 10.26 | 9.42 | 12.91 | 10.36 | 9.12 | 9.90 | |
|  | *9* | 7.68 | 8.58 | 9.55 | 8.98 | 9.66 | 9.74 | 10.32 | 9.29 | 14.21 | 9.83 | 10.60 | 10.08 | |
|  | *10* | 7.31 | 11.96 | 8.98 | 11.76 | 11.07 | 9.21 | 7.56 | 8.79 | 12.92 | 10.55 | 11.27 | 10.41 | |
|  | *11* | 10.07 | 11.96 | 8.98 | 11.76 | 11.87 | 12.19 | 12.19 | 13.67 | 15.23 | 10.55 | 11.27 | 11.97 | |
|  | *12* | 6.20 | 7.43 | 7.10 | 7.22 | 7.80 | 7.20 | 7.20 | 7.60 | 7.23 | 7.50 | 7.40 | 7.37 | |
|  | *13* | 7.86 | 8.69 | 10.30 | 8.49 | 8.85 | 7.70 | 10.46 | 9.92 | 11.76 | 9.16 | 8.29 | 9.36 | |
| Sub-5 | *1* | 4.80 | 6.04 | 4.38 | 5.08 | 4.95 | 5.85 | 5.78 | 5.32 | 6.89 | 4.28 | 4.13 | 5.27 | |
|  | *2* | 4.67 | 4.00 | 3.53 | 5.44 | 5.29 | 5.83 | 5.27 | 4.88 | 5.29 | 4.66 | 4.73 | 4.89 | |
| Sub-6 | *1* | 6.42 | 7.77 | 5.94 | 6.86 | 6.04 | 7.45 | 7.92 | 5.97 | 8.51 | 7.66 | 7.22 | 7.13 | |
| Sub-7 | *1* | 9.15 | 11.64 | 9.76 | 10.18 | 11.54 | 8.88 | 12.60 | 9.70 | 12.17 | 10.31 | 10.18 | 10.70 | |
| Sub-8 | *1* | 4.63 | 4.81 | 4.80 | 6.74 | 6.00 | 4.92 | 5.48 | 6.21 | 5.14 | 4.93 | 5.00 | 5.40 | |
|  | *2* | 4.56 | 4.04 | 4.98 | 5.96 | 5.46 | 4.87 | 5.34 | 4.73 | 5.48 | 4.59 | 6.04 | 5.15 | |
|  | *3* | 6.53 | 6.42 | 6.76 | 6.85 | 7.16 | 7.10 | 6.27 | 6.47 | 6.39 | 7.04 | 6.68 | 6.71 | |
|  | *4* | 6.03 | 5.89 | 5.99 | 6.93 | 6.03 | 7.04 | 5.79 | 5.53 | 7.19 | 5.62 | 6.47 | 6.25 | |
|  | *5* | 5.61 | 5.68 | 5.67 | 6.58 | 6.24 | 6.14 | 5.74 | 5.88 | 6.58 | 5.46 | 5.28 | 5.93 | |
|  | *6* | 4.61 | 4.22 | 4.57 | 5.00 | 4.93 | 5.49 | 4.90 | 3.87 | 4.88 | 3.84 | 3.50 | 4.52 | |
|  | *7* | 5.71 | 5.95 | 5.67 | 6.69 | 6.42 | 6.53 | 6.04 | 6.64 | 6.79 | 5.91 | 5.21 | 6.19 | |
|  | *8* | 7.45 | 8.05 | 7.84 | 8.04 | 8.49 | 7.91 | 7.40 | 8.09 | 8.33 | 7.96 | 8.03 | 8.01 | |
|  | *9* | 4.44 | 4.57 | 4.40 | 4.82 | 4.96 | 5.42 | 5.09 | 5.14 | 5.23 | 4.35 | 4.49 | 4.85 | |
| Sub-9 | *1* | 9.60 | 10.87 | 10.50 | 8.66 | 9.95 | 8.46 | 9.69 | 9.58 | 10.91 | 9.01 | 9.94 | 9.76 | |
| Sub-10 | *1* | 7.04 | 9.04 | 7.06 | 7.33 | 8.32 | 8.98 | 8.30 | 9.28 | 9.21 | 9.29 | 9.83 | 8.66 | |
|  | *2* | 8.62 | 9.10 | 8.99 | 11.02 | 9.47 | 12.38 | 9.77 | 8.73 | 8.98 | 10.42 | 10.52 | 9.94 | |
|  | *3* | 6.80 | 7.92 | 8.13 | 6.76 | 7.68 | 9.09 | 8.99 | 7.72 | 9.16 | 6.17 | 8.41 | 8.00 | |
| Sub-11 | *1* | 6.09 | 10.95 | 13.17 | 10.04 | 8.14 | 7.49 | 8.62 | 9.43 | 9.18 | 8.89 | 8.24 | 9.42 | |
|  | *2* | 6.79 | 9.16 | 9.58 | 8.47 | 9.19 | 7.15 | 8.49 | 9.10 | 8.76 | 8.85 | 9.32 | 8.81 | |
|  | *3* | 4.43 | 4.54 | 5.37 | 5.55 | 4.59 | 5.33 | 4.31 | 4.30 | 6.24 | 5.82 | 5.41 | 5.15 | |
|  | *4* | 6.53 | 7.83 | 8.60 | 9.85 | 7.54 | 9.89 | 8.64 | 6.65 | 8.87 | 6.72 | 7.07 | 8.17 | |
|  | *5* | 6.40 | 6.42 | 7.74 | 5.96 | 8.99 | 10.02 | 6.11 | 5.13 | 8.27 | 7.97 | 4.45 | 7.11 | |
|  | *6* | 8.29 | 9.55 | 11.42 | 9.22 | 9.17 | 8.36 | 8.46 | 10.24 | 10.13 | 9.41 | 8.07 | 9.40 | |
|  | *7* | 6.48 | 7.91 | 8.70 | 9.19 | 7.23 | 7.30 | 6.85 | 7.27 | 7.87 | 7.18 | 8.37 | 7.79 | |
|  | *8* | 6.11 | 5.75 | 6.57 | 5.83 | 5.07 | 6.13 | 5.55 | 5.96 | 5.86 | 5.71 | 6.58 | 5.90 | |
| Sub-12 | *1* | 4.89 | 4.76 | 4.62 | 4.67 | 4.89 | 5.58 | 5.37 | 4.15 | 4.67 | 4.41 | 4.27 | 4.74 | |
| Sub-13 | *1* | 8.55 | 9.71 | 7.90 | 7.77 | 9.17 | 9.00 | 7.81 | 8.21 | 7.59 | 9.10 | 8.52 | 8.48 | |
|  | *2* | 13.23 | 14.13 | 13.79 | 13.74 | 14.15 | 14.86 | 19.16 | 14.38 | 11.63 | 15.84 | 11.63 | 14.33 | |
|  | *3* | 13.56 | 15.81 | 12.21 | 12.72 | 15.29 | 12.77 | 14.65 | 18.05 | 13.18 | 14.34 | 13.86 | 14.29 | |
|  | *4* | 16.44 | 19.01 | 17.63 | 17.27 | 18.46 | 19.81 | 18.62 | 19.94 | 17.23 | 17.09 | 17.88 | 18.29 | |
|  | *5* | 14.80 | 16.93 | 17.21 | 16.81 | 15.82 | 15.55 | 17.29 | 16.39 | 18.60 | 15.97 | 17.83 | 16.84 | |
|  | *6* | 12.71 | 14.55 | 13.84 | 15.51 | 14.67 | 14.31 | 16.31 | 13.16 | 13.23 | 13.16 | 13.27 | 14.20 | |
|  | *7* | 10.33 | 9.91 | 12.16 | 11.95 | 13.62 | 14.57 | 11.83 | 10.93 | 13.23 | 11.88 | 12.89 | 12.30 | |
|  | *8* | 9.91 | 12.66 | 12.18 | 10.32 | 10.32 | 15.09 | 12.05 | 15.94 | 12.34 | 11.01 | 12.17 | 12.41 | |
|  | *9* | 6.90 | 6.00 | 6.56 | 6.46 | 7.49 | 6.06 | 6.95 | 5.26 | 7.37 | 6.68 | 6.89 | 6.57 | |
|  | *10* | 15.00 | 16.78 | 18.24 | 15.57 | 19.10 | 14.70 | 18.38 | 15.23 | 16.78 | 15.20 | 16.11 | 16.61 | |
|  | *11* | 7.38 | 6.41 | 6.28 | 6.26 | 6.68 | 6.37 | 7.71 | 7.15 | 8.07 | 5.76 | 7.44 | 6.81 | |
|  | *12* | 14.27 | 16.41 | 14.53 | 17.51 | 17.96 | 16.52 | 20.01 | 17.49 | 14.70 | 17.55 | 13.80 | 16.65 | |
|  | *13* | 15.10 | 16.28 | 17.38 | 19.13 | 15.98 | 18.46 | 16.86 | 16.10 | 17.64 | 15.28 | 15.15 | 16.83 | |
| Sub-14 | *1* | 11.68 | 13.56 | 14.72 | 14.47 | 15.83 | 16.15 | 17.02 | 16.02 | 12.47 | 13.72 | 15.11 | 14.91 | |
|  | *2* | 7.86 | 7.58 | 9.14 | 8.45 | 9.29 | 8.46 | 9.93 | 8.29 | 8.67 | 7.71 | 8.63 | 8.62 | |
|  | *3* | 10.17 | 14.53 | 13.74 | 13.04 | 13.73 | 15.58 | 16.17 | 16.04 | 12.99 | 13.84 | 12.92 | 14.26 | |
|  | *4* | 25.88 | 29.83 | 28.51 | 30.89 | 31.01 | 28.60 | 30.21 | 30.08 | 31.82 | 29.27 | 30.58 | 30.08 | |
|  | *5* | 7.68 | 8.33 | 7.23 | 9.43 | 9.00 | 13.21 | 13.53 | 9.87 | 8.75 | 8.76 | 10.00 | 9.81 | |
|  | *6* | 7.34 | 7..86 | 7.46 | 9.30 | 7.62 | 8.33 | 8.46 | 7.46 | 8.03 | 9.13 | 8.21 | 8.22 | |
|  | *7* | 24.70 | 32.00 | 30.89 | 28.19 | 29.66 | 31.46 | 35.55 | 32.55 | 29.45 | 32.38 | 28.53 | 31.07 | |
|  | *8* | 19.64 | 26.50 | 24.55 | 25.44 | 26.75 | 31.75 | 29.37 | 28.19 | 25.39 | 26.09 | 27.88 | 27.19 | |
|  | *9* | 9.26 | 11.77 | 10.25 | 14.51 | 15.75 | 20.77 | 16.52 | 13.60 | 12.75 | 11.75 | 9.78 | 13.75 | |
|  | *10* | 8.26 | 10.56 | 8.30 | 10.97 | 10.38 | 10.01 | 11.79 | 10.33 | 8.83 | 8.04 | 8.67 | 9.79 | |
|  | *11* | 17.71 | 21.34 | 17.02 | 22.62 | 23.37 | 21.93 | 26.97 | 18.60 | 21.15 | 22.57 | 16.81 | 21.24 | |
|  | *12* | 13.40 | 14.17 | 12.89 | 13.87 | 14.68 | 15.93 | 15.58 | 15.67 | 14.02 | 13.70 | 14.62 | 14.51 | |
|  | *13* | 13.03 | 11.41 | 11.99 | 13.88 | 11.59 | 13.01 | 16.31 | 13.31 | 14.68 | 13.30 | 12.34 | 13.18 | |
| Sub-15 | *1* | 7.87 | 9.00 | 8.74 | 8.83 | 8.91 | 8.82 | 8.60 | 9.05 | 9.38 | 8.89 | 8.82 | 8.90 | |
| Sub-16 | *1* | 6.90 | 8.49 | 9.09 | 7.83 | 9.43 | 7.61 | 9.89 | 8.05 | 8.60 | 9.70 | 9.34 | 8.80 | |
|  | *2* | 4.33 | 4.26 | 4.97 | 5.75 | 5.11 | 4.94 | 5.98 | 5.11 | 3.97 | 4.60 | 3.93 | 4.86 | |
|  | *3* | 6.40 | 6.61 | 7.46 | 6.98 | 7.12 | 7.18 | 9.16 | 7.85 | 6.86 | 7.13 | 6.52 | 7.29 | |
|  | *4* | 7.37 | 8.12 | 8.04 | 9.07 | 8.08 | 9.93 | 9.41 | 9.74 | 7.60 | 7.92 | 7.55 | 8.55 | |

**Supplementary Table 3:**

**Supplementary Table 3.** Ungated SUVmean, variations of SUVmean between respiratory phases, and averaged SUVmean averaged over the 10 phases for all the 89 lesions in this study.

| **Subject (Sub)** | ***lesion*** | **Ungated SUVmean** | **SUVmean across respiratory phases** | | | | | | | | | | **Averaged SUVmean over the 10 phases** |
| --- | --- | --- | --- | --- | --- | --- | --- | --- | --- | --- | --- | --- | --- |
|  |  |  | **10%** | **20%** | **30%** | **40%** | **50%** | **60%** | **70%** | **80%** | **90%** | **100%** |  |
| Sub-1 | *1* | 9.67 | 12.57 | 12.68 | 12.42 | 12.24 | 11.92 | 11.73 | 11.94 | 12.57 | 12.43 | 12.45 | 12.30 |
|  | *2* | 6.21 | 7.15 | 7.45 | 7.08 | 7.14 | 7.14 | 7.28 | 7.07 | 7.41 | 7.01 | 7.01 | 7.17 |
|  | *3* | 6.55 | 7.31 | 6.93 | 6.42 | 6.89 | 6.50 | 6.76 | 6.46 | 6.53 | 6.65 | 6.97 | 6.74 |
|  | *4* | 5.70 | 6.97 | 7.14 | 6.65 | 6.60 | 6.87 | 6.72 | 6.61 | 6.80 | 6.58 | 6.47 | 6.74 |
|  | *5* | 5.37 | 5.93 | 6.53 | 6.54 | 6.02 | 6.15 | 5.63 | 6.07 | 6.31 | 6.09 | 6.21 | 6.15 |
| Sub-2 | *1* | 5.19 | 6.82 | 7.28 | 6.92 | 7.08 | 7.26 | 7.18 | 6.86 | 7.45 | 7.57 | 7.46 | 7.19 |
|  | *2* | 4.92 | 7.75 | 7.99 | 8.11 | 8.54 | 8.27 | 8.27 | 8.04 | 8.62 | 8.43 | 8.28 | 8.23 |
|  | *3* | 5.07 | 7.43 | 8.11 | 6.75 | 6.57 | 6.52 | 6.32 | 6.39 | 6.80 | 6.71 | 6.22 | 6.78 |
|  | *4* | 4.60 | 5.57 | 5.60 | 5.56 | 5.52 | 5.35 | 5.58 | 5.30 | 5.70 | 5.76 | 5.52 | 5.55 |
|  | *5* | 4.20 | 5.65 | 5.15 | 5.21 | 5.34 | 5.23 | 5.62 | 5.44 | 5.62 | 5.49 | 5.60 | 5.44 |
|  | *6* | 3.72 | 4.62 | 4.46 | 4.36 | 4.62 | 4.44 | 4.45 | 4.45 | 4.57 | 4.54 | 4.43 | 4.49 |
|  | *7* | 5.03 | 5.83 | 4.19 | 6.22 | 6.41 | 6.24 | 6.40 | 6.32 | 6.42 | 6.43 | 6.15 | 6.06 |
|  | *8* | 5.79 | 6.78 | 6.70 | 6.69 | 5.84 | 6.71 | 6.38 | 6.36 | 6.63 | 6.28 | 6.38 | 6.48 |
|  | *9* | 4.55 | 5.55 | 5.30 | 5.61 | 5.24 | 5.68 | 5.35 | 5.40 | 5.87 | 5.16 | 5.63 | 5.48 |
|  | *10* | 5.70 | 6.40 | 6.85 | 6.77 | 6.82 | 6.92 | 6.86 | 6.75 | 6.94 | 6.69 | 6.94 | 6.79 |
|  | *11* | 5.04 | 5.84 | 5.82 | 5.83 | 5.87 | 5.90 | 6.01 | 5.89 | 5.90 | 6.02 | 6.04 | 5.91 |
|  | *12* | 4.19 | 4.95 | 4.86 | 4.94 | 5.01 | 5.16 | 5.31 | 5.04 | 5.73 | 5.53 | 5.36 | 5.19 |
| Sub-3 | *1* | 5.01 | 5.48 | 5.33 | 5.45 | 5.43 | 5.43 | 5.34 | 5.42 | 5.38 | 5.35 | 5.34 | 5.40 |
|  | *2* | 4.85 | 5.45 | 5.59 | 5.89 | 5.50 | 5.73 | 5.77 | 5.66 | 5.74 | 5.61 | 5.67 | 5.66 |
| Sub-4 | *1* | 4.57 | 5.63 | 5.33 | 5.34 | 5.10 | 6.06 | 5.37 | 5.64 | 5.47 | 5.56 | 5.98 | 5.55 |
|  | *2* | 5.70 | 7.02 | 7.88 | 7.49 | 7.15 | 7.44 | 7.40 | 7.73 | 8.14 | 7.64 | 7.79 | 7.57 |
|  | *3* | 4.43 | 4.92 | 4.69 | 5.73 | 5.40 | 5.73 | 5.45 | 4.91 | 5.67 | 6.13 | 5.62 | 5.43 |
|  | *4* | 4.57 | 5.91 | 6.02 | 5.90 | 6.02 | 5.65 | 5.96 | 5.87 | 6.20 | 5.76 | 5.73 | 5.90 |
|  | *5* | 7.41 | 7.60 | 7.07 | 8.53 | 8.24 | 8.23 | 8.24 | 8.12 | 8.14 | 8.13 | 7.86 | 8.02 |
|  | *6* | 4.61 | 4.84 | 4.39 | 5.55 | 5.11 | 5.89 | 5.89 | 6.22 | 6.77 | 6.16 | 5.18 | 5.60 |
|  | *7* | 4.43 | 4.68 | 4.37 | 5.25 | 4.39 | 4.73 | 4.41 | 4.28 | 5.14 | 4.95 | 4.59 | 4.68 |
|  | *8* | 4.88 | 5.72 | 6.15 | 5.88 | 6.32 | 6.42 | 6.40 | 6.43 | 6.68 | 6.50 | 6.22 | 6.27 |
|  | *9* | 5.48 | 5.72 | 6.33 | 6.11 | 6.34 | 6.62 | 6.64 | 6.52 | 7.02 | 6.54 | 6.70 | 6.45 |
|  | *10* | 5.29 | 5.80 | 5.78 | 6.35 | 6.17 | 5.68 | 5.54 | 6.07 | 6.25 | 6.26 | 6.24 | 6.01 |
|  | *11* | 5.45 | 5.80 | 5.43 | 6.04 | 6.48 | 6.13 | 6.05 | 6.41 | 6.51 | 6.12 | 5.81 | 6.08 |
|  | *12* | 5.15 | 6.46 | 6.50 | 6.31 | 6.24 | 6.71 | 6.53 | 6.47 | 6.41 | 6.70 | 6.80 | 6.51 |
|  | *13* | 5.67 | 5.91 | 6.02 | 5.90 | 6.02 | 5.65 | 5.96 | 5.87 | 6.20 | 5.76 | 5.73 | 5.90 |
| Sub-5 | *1* | 2.96 | 3.38 | 3.21 | 3.44 | 3.75 | 3.90 | 3.84 | 3.74 | 3.54 | 3.38 | 3.29 | 3.55 |
|  | *2* | 2.77 | 3.14 | 2.97 | 3.42 | 3.24 | 3.63 | 3.50 | 3.49 | 3.47 | 3.33 | 3.18 | 3.34 |
| Sub-6 | *1* | 4.15 | 5.06 | 4.57 | 4.98 | 4.79 | 4.92 | 4.95 | 4.57 | 4.86 | 4.83 | 4.55 | 4.81 |
| Sub-7 | *1* | 6.34 | 7.98 | 7.70 | 7.50 | 8.17 | 7.25 | 8.01 | 7.63 | 8.09 | 7.96 | 7.86 | 7.82 |
| Sub-8 | *1* | 3.69 | 4.18 | 4.21 | 4.28 | 4.41 | 4.34 | 4.36 | 4.36 | 4.25 | 4.24 | 4.23 | 4.29 |
|  | *2* | 3.42 | 3.50 | 3.85 | 3.88 | 4.14 | 3.70 | 3.80 | 3.76 | 3.85 | 3.64 | 3.88 | 3.80 |
|  | *3* | 4.01 | 4.68 | 4.81 | 4.68 | 4.55 | 4.87 | 4.78 | 4.78 | 4.79 | 4.88 | 4.75 | 4.76 |
|  | *4* | 4.02 | 4.70 | 4.79 | 4.88 | 4.66 | 4.83 | 4.66 | 4.46 | 4.76 | 4.54 | 4.59 | 4.69 |
|  | *5* | 4.05 | 4.43 | 4.91 | 5.01 | 4.75 | 4.68 | 4.61 | 4.66 | 4.84 | 4.51 | 4.53 | 4.69 |
|  | *6* | 3.96 | 3.50 | 3.66 | 3.97 | 4.04 | 4.33 | 4.13 | 3.46 | 3.87 | 3.49 | 3.37 | 3.78 |
|  | *7* | 4.10 | 4.51 | 4.43 | 4.84 | 4.83 | 4.77 | 4.59 | 4.81 | 4.77 | 4.54 | 4.34 | 4.64 |
|  | *8* | 4.45 | 4.95 | 4.92 | 5.01 | 5.21 | 4.92 | 4.89 | 4.94 | 4.97 | 5.00 | 4.92 | 4.97 |
|  | *9* | 3.49 | 3.78 | 3.77 | 3.90 | 3.89 | 3.99 | 3.94 | 4.02 | 3.95 | 3.70 | 3.68 | 3.86 |
| Sub-9 | *1* | 6.16 | 6.26 | 6.31 | 6.12 | 6.26 | 5.93 | 6.22 | 6.73 | 6.83 | 5.28 | 6.42 | 6.24 |
| Sub-10 | *1* | 4.92 | 4.86 | 4.61 | 4.57 | 4.60 | 4.91 | 5.05 | 4.88 | 5.39 | 4.73 | 4.81 | 4.84 |
|  | *2* | 5.82 | 5.90 | 5.84 | 6.03 | 6.15 | 5.92 | 6.02 | 5.94 | 5.99 | 6.07 | 5.87 | 5.97 |
|  | *3* | 5.37 | 5.45 | 5.88 | 5.44 | 5.73 | 5.80 | 6.20 | 5.49 | 6.09 | 5.02 | 5.97 | 5.71 |
| Sub-11 | *1* | 3.85 | 5.25 | 5.14 | 5.22 | 5.16 | 5.14 | 5.18 | 5.40 | 5.24 | 5.26 | 5.20 | 5.22 |
|  | *2* | 4.48 | 5.57 | 5.85 | 5.58 | 5.58 | 5.42 | 5.44 | 5.80 | 5.41 | 5.64 | 6.00 | 5.63 |
|  | *3* | 3.32 | 3.53 | 4.24 | 4.33 | 3.93 | 4.23 | 3.66 | 3.35 | 4.38 | 4.34 | 4.17 | 4.02 |
|  | *4* | 3.90 | 5.50 | 5.47 | 5.84 | 5.47 | 5.45 | 5.69 | 4.89 | 5.32 | 5.06 | 5.23 | 5.39 |
|  | *5* | 3.93 | 4.76 | 5.31 | 4.60 | 5.15 | 5.43 | 4.75 | 4.23 | 5.40 | 5.30 | 3.78 | 4.87 |
|  | *6* | 4.30 | 5.63 | 5.74 | 5.56 | 5.56 | 5.35 | 5.41 | 5.42 | 5.80 | 5.78 | 5.54 | 5.58 |
|  | *7* | 4.50 | 5.23 | 5.21 | 5.07 | 5.12 | 5.48 | 5.25 | 5.15 | 5.12 | 5.09 | 5.49 | 5.22 |
|  | *8* | 3.96 | 4.44 | 4.45 | 4.26 | 4.13 | 4.38 | 4.16 | 4.56 | 4.21 | 4.28 | 4.29 | 4.32 |
| Sub-12 | *1* | 3.97 | 3.74 | 3.65 | 3.82 | 3.86 | 3.80 | 4.09 | 3.49 | 3.90 | 3.71 | 3.55 | 3.76 |
| Sub-13 | *1* | 5.57 | 5.88 | 5.61 | 5.60 | 6.04 | 6.00 | 5.50 | 5.91 | 5.69 | 5.91 | 6.01 | 5.82 |
|  | *2* | 7.12 | 11.56 | 9.93 | 10.58 | 11.71 | 11.58 | 12.34 | 11.68 | 9.52 | 12.07 | 9.47 | 11.04 |
|  | *3* | 7.36 | 10.03 | 8.87 | 8.96 | 10.61 | 9.37 | 10.33 | 10.68 | 9.65 | 10.33 | 9.55 | 9.84 |
|  | *4* | 9.12 | 11.66 | 11.57 | 11.58 | 11.77 | 11.70 | 11.75 | 11.63 | 11.38 | 11.59 | 11.48 | 11.61 |
|  | *5* | 8.58 | 10.88 | 10.99 | 10.97 | 10.71 | 10.50 | 10.91 | 10.85 | 11.18 | 10.87 | 11.12 | 10.90 |
|  | *6* | 7.20 | 8.89 | 10.16 | 10.60 | 10.40 | 9.99 | 10.43 | 10.40 | 9.43 | 9.05 | 9.44 | 9.88 |
|  | *7* | 6.99 | 8.19 | 8.62 | 7.69 | 8.45 | 8.79 | 8.81 | 8.39 | 8.50 | 8.78 | 8.59 | 8.48 |
|  | *8* | 5.43 | 7.92 | 7.89 | 7.76 | 7.76 | 7.43 | 8.36 | 8.97 | 8.26 | 7.63 | 8.77 | 8.08 |
|  | *9* | 4.85 | 5.50 | 5.40 | 5.43 | 5.88 | 5.37 | 5.54 | 5.04 | 5.99 | 5.37 | 5.71 | 5.52 |
|  | *10* | 7.53 | 10.71 | 11.10 | 10.39 | 11.38 | 10.28 | 11.11 | 10.54 | 11.18 | 10.57 | 10.57 | 10.78 |
|  | *11* | 5.00 | 5.36 | 4.79 | 5.41 | 5.65 | 4.93 | 6.51 | 6.19 | 6.32 | 4.86 | 6.04 | 5.61 |
|  | *12* | 8.29 | 11.87 | 10.93 | 11.11 | 10.89 | 11.21 | 11.62 | 11.15 | 10.84 | 10.97 | 10.61 | 11.12 |
|  | *13* | 8.60 | 11.12 | 10.92 | 10.60 | 11.43 | 11.60 | 11.23 | 11.46 | 11.45 | 11.54 | 11.04 | 11.24 |
| Sub-14 | *1* | 7.75 | 11.39 | 12.48 | 12.51 | 13.49 | 13.57 | 14.15 | 13.19 | 10.75 | 11.91 | 12.64 | 12.61 |
|  | *2* | 5.28 | 6.61 | 7.96 | 7.50 | 8.07 | 7.70 | 8.71 | 7.29 | 7.51 | 6.97 | 7.96 | 7.63 |
|  | *3* | 7.41 | 9.62 | 10.53 | 9.98 | 10.65 | 10.72 | 10.40 | 11.14 | 9.62 | 10.18 | 9.23 | 10.21 |
|  | *4* | 14.86 | 16.31 | 16.01 | 16.43 | 16.43 | 16.58 | 16.52 | 16.60 | 16.09 | 16.37 | 16.62 | 16.40 |
|  | *5* | 5.51 | 7.29 | 5.77 | 8.13 | 7.14 | 10.26 | 10.52 | 8.28 | 7.00 | 6.96 | 8.04 | 7.94 |
|  | *6* | 4.99 | 5.43 | 5.42 | 7.18 | 5.31 | 5.64 | 5.89 | 5.43 | 5.68 | 5.97 | 5.69 | 5.76 |
|  | *7* | 12.08 | 28.68 | 17.46 | 17.24 | 17.58 | 17.71 | 18.34 | 18.08 | 17.64 | 17.99 | 17.54 | 18.83 |
|  | *8* | 9.72 | 15.45 | 15.13 | 15.67 | 15.59 | 17.69 | 16.11 | 15.89 | 15.40 | 14.02 | 14.76 | 15.57 |
|  | *9* | 5.83 | 9.92 | 8.98 | 10.98 | 12.54 | 10.07 | 8.77 | 11.20 | 10.77 | 8.18 | 7.78 | 9.92 |
|  | *10* | 5.62 | 8.94 | 6.09 | 9.38 | 8.96 | 8.77 | 9.79 | 7.52 | 6.66 | 6.13 | 6.39 | 7.86 |
|  | *11* | 8.31 | 12.67 | 11.96 | 13.54 | 13.52 | 12.69 | 14.52 | 12.45 | 12.66 | 12.91 | 11.92 | 12.88 |
|  | *12* | 8.35 | 10.07 | 9.55 | 9.99 | 10.30 | 10.30 | 10.31 | 10.33 | 9.87 | 9.86 | 10.13 | 10.07 |
|  | *13* | 6.78 | 8.90 | 9.18 | 9.92 | 8.99 | 9.64 | 10.31 | 9.63 | 9.94 | 9.47 | 9.37 | 9.54 |
| Sub-15 | *1* | 4.13 | 4.36 | 4.39 | 4.37 | 4.41 | 4.46 | 4.44 | 4.37 | 4.42 | 4.42 | 4.36 | 4.40 |
| Sub-16 | *1* | 5.04 | 6.44 | 6.44 | 6.22 | 6.82 | 5.53 | 7.16 | 6.24 | 6.31 | 7.12 | 6.83 | 6.51 |
|  | *2* | 3.52 | 3.79 | 4.24 | 4.69 | 4.45 | 4.14 | 4.59 | 4.35 | 3.67 | 4.17 | 3.73 | 4.18 |
|  | *3* | 4.13 | 5.09 | 5.25 | 5.40 | 5.39 | 5.45 | 5.82 | 5.68 | 5.43 | 5.34 | 5.17 | 5.40 |
|  | *4* | 4.79 | 5.60 | 5.92 | 5.97 | 5.70 | 6.06 | 5.99 | 6.26 | 5.87 | 5.91 | 5.55 | 5.88 |

**Supplementary Table 4:**

**Supplementary Table 4.** Ungated TLG, variations of SUV TLG between respiratory phases, and averaged TLG averaged over the 10 phases for all the 89 lesions in this study.

| **Subject (Sub)** | ***lesion*** | **Ungated TLG** | **TLG across respiratory phases** | | | | | | | | | | **Averaged TLG over the 10 phases** |
| --- | --- | --- | --- | --- | --- | --- | --- | --- | --- | --- | --- | --- | --- |
|  |  |  | **10%** | **20%** | **30%** | **40%** | **50%** | **60%** | **70%** | **80%** | **90%** | **100%** |  |
| Sub-1 | *1* | 27.10 | 24.35 | 24.03 | 24.45 | 24.64 | 25.23 | 25.88 | 25.38 | 24.07 | 24.55 | 24.52 | 24.71 |
|  | *2* | 26.66 | 28.96 | 25.45 | 27.85 | 28.00 | 26.42 | 26.08 | 27.51 | 26.22 | 28.74 | 28.96 | 27.42 |
|  | *3* | 6.33 | 3.23 | 4.47 | 5.39 | 4.18 | 5.02 | 3.97 | 5.23 | 5.12 | 4.39 | 4.19 | 4.52 |
|  | *4* | 12.53 | 14.08 | 10.81 | 13.69 | 13.50 | 13.17 | 13.07 | 13.54 | 14.68 | 14.53 | 17.02 | 13.81 |
|  | *5* | 3.49 | 4.09 | 2.80 | 2.95 | 3.31 | 3.51 | 3.35 | 3.37 | 3.37 | 3.61 | 3.85 | 3.42 |
| Sub-2 | *1* | 3.13 | 5.76 | 4.53 | 5.11 | 4.95 | 5.63 | 4.88 | 4.46 | 5.09 | 4.95 | 5.09 | 5.05 |
|  | *2* | 10.77 | 13.09 | 11.84 | 11.40 | 11.03 | 11.36 | 11.76 | 11.07 | 10.71 | 10.68 | 11.46 | 11.44 |
|  | *3* | 12.39 | 10.04 | 9.66 | 12.71 | 13.01 | 12.78 | 14.22 | 13.71 | 12.83 | 12.46 | 14.48 | 12.59 |
|  | *4* | 27.54 | 29.21 | 31.43 | 32.21 | 31.23 | 33.07 | 29.83 | 33.82 | 31.13 | 29.33 | 31.07 | 31.23 |
|  | *5* | 6.75 | 8.00 | 7.93 | 8.49 | 7.86 | 8.38 | 7.70 | 7.43 | 8.33 | 7.84 | 8.10 | 8.01 |
|  | *6* | 5.22 | 4.24 | 4.08 | 4.18 | 4.37 | 3.91 | 4.13 | 3.96 | 4.20 | 3.99 | 3.61 | 4.07 |
|  | *7* | 17.45 | 22.25 | 19.86 | 20.49 | 19.31 | 19.91 | 19.65 | 19.50 | 19.66 | 18.78 | 19.45 | 19.89 |
|  | *8* | 29.76 | 24.27 | 24.49 | 24.52 | 25.79 | 24.65 | 26.59 | 26.95 | 25.76 | 26.99 | 26.64 | 25.67 |
|  | *9* | 8.78 | 10.31 | 10.02 | 10.19 | 10.23 | 9.17 | 9.06 | 9.90 | 9.67 | 9.25 | 9.57 | 9.74 |
|  | *10* | 15.82 | 19.26 | 17.61 | 17.50 | 17.14 | 16.93 | 17.46 | 17.54 | 17.43 | 18.68 | 16.98 | 17.65 |
|  | *11* | 11.72 | 12.94 | 13.30 | 12.94 | 12.76 | 12.56 | 11.45 | 12.26 | 12.47 | 12.08 | 11.80 | 12.46 |
|  | *12* | 3.56 | 3.90 | 3.68 | 3.85 | 4.58 | 4.43 | 3.55 | 3.32 | 3.25 | 3.70 | 3.33 | 3.76 |
| Sub-3 | *1* | 87.48 | 102.19 | 109.40 | 102.93 | 112.82 | 105.66 | 111.20 | 108.16 | 109.21 | 114.94 | 109.65 | 108.62 |
|  | *2* | 95.72 | 108.77 | 107.76 | 87.83 | 106.64 | 101.09 | 103.49 | 106.89 | 110.13 | 104.05 | 107.08 | 104.37 |
| Sub-4 | *1* | 16.67 | 14.88 | 24.35 | 22.53 | 28.09 | 16.03 | 23.10 | 22.91 | 25.28 | 23.99 | 19.87 | 22.10 |
|  | *2* | 7.10 | 8.15 | 8.19 | 8.28 | 8.20 | 9.05 | 9.25 | 8.01 | 8.12 | 7.65 | 8.72 | 8.36 |
|  | *3* | 2.48 | 1.97 | 3.64 | 2.47 | 2.63 | 1.98 | 2.24 | 1.61 | 2.01 | 2.15 | 2.26 | 2.30 |
|  | *4* | 13.59 | 9.68 | 9.39 | 10.01 | 9.94 | 11.89 | 13.42 | 12.15 | 12.12 | 13.41 | 8.99 | 11.10 |
|  | *5* | 18.92 | 24.72 | 24.06 | 22.18 | 23.62 | 23.45 | 23.85 | 24.29 | 23.85 | 23.68 | 24.94 | 23.86 |
|  | *6* | 4.49 | 3.12 | 1.77 | 4.23 | 5.51 | 7.99 | 8.72 | 8.41 | 7.33 | 7.41 | 5.45 | 5.99 |
|  | *7* | 4.48 | 3.57 | 4.10 | 3.19 | 3.43 | 3.82 | 3.49 | 4.23 | 3.81 | 4.06 | 3.20 | 3.69 |
|  | *8* | 7.36 | 7.40 | 8.17 | 8.44 | 9.28 | 9.11 | 9.35 | 8.69 | 9.34 | 9.06 | 8.20 | 8.70 |
|  | *9* | 6.89 | 7.40 | 10.76 | 11.49 | 11.46 | 11.42 | 11.48 | 11.90 | 12.07 | 11.75 | 11.38 | 11.11 |
|  | *10* | 7.03 | 9.32 | 7.66 | 8.47 | 8.68 | 8.82 | 8.44 | 7.70 | 9.40 | 8.49 | 8.15 | 8.51 |
|  | *11* | 15.59 | 13.37 | 13.99 | 13.13 | 16.65 | 18.40 | 17.95 | 17.50 | 19.52 | 15.35 | 11.60 | 15.75 |
|  | *12* | 2.32 | 2.10 | 2.22 | 2.31 | 2.13 | 2.28 | 2.37 | 2.50 | 2.41 | 2.37 | 2.30 | 2.30 |
|  | *13* | 7.37 | 9.68 | 9.39 | 10.01 | 9.94 | 11.89 | 13.42 | 12.15 | 12.12 | 13.41 | 8.99 | 11.10 |
| Sub-5 | *1* | 12.06 | 8.95 | 9.75 | 9.08 | 6.48 | 6.02 | 6.45 | 6.41 | 8.24 | 7.69 | 8.42 | 7.75 |
|  | *2* | 7.22 | 4.78 | 5.40 | 5.62 | 7.02 | 4.90 | 5.28 | 5.14 | 5.63 | 5.85 | 5.94 | 5.56 |
| Sub-6 | *1* | 9.87 | 6.53 | 7.59 | 7.10 | 7.41 | 7.60 | 8.09 | 7.92 | 8.63 | 7.83 | 8.02 | 7.67 |
| Sub-7 | *1* | 14.01 | 13.24 | 14.28 | 15.67 | 13.19 | 16.30 | 14.24 | 13.72 | 13.69 | 12.58 | 12.41 | 13.93 |
| Sub-8 | *1* | 8.38 | 4.02 | 5.75 | 4.01 | 4.60 | 3.91 | 3.68 | 4.55 | 5.25 | 5.42 | 3.84 | 4.50 |
|  | *2* | 5.79 | 3.93 | 3.37 | 3.22 | 2.22 | 4.20 | 3.84 | 3.87 | 3.25 | 4.41 | 3.65 | 3.60 |
|  | *3* | 13.65 | 9.03 | 8.42 | 9.34 | 9.87 | 8.05 | 8.36 | 8.46 | 8.71 | 8.14 | 8.55 | 8.69 |
|  | *4* | 16.76 | 7.47 | 7.08 | 7.22 | 8.77 | 7.77 | 9.30 | 9.75 | 8.36 | 8.55 | 9.12 | 8.34 |
|  | *5* | 11.36 | 8.45 | 6.04 | 6.36 | 8.52 | 8.98 | 9.37 | 9.72 | 7.15 | 9.48 | 6.35 | 8.04 |
|  | *6* | 1.85 | 5.03 | 2.98 | 1.36 | 0.96 | 0.89 | 1.01 | 2.90 | 1.69 | 1.82 | 1.50 | 2.01 |
|  | *7* | 13.97 | 10.77 | 11.68 | 9.46 | 9.21 | 9.22 | 10.31 | 9.17 | 9.75 | 9.96 | 10.92 | 10.05 |
|  | *8* | 44.99 | 44.63 | 45.67 | 43.92 | 40.13 | 45.42 | 47.67 | 45.82 | 45.53 | 43.77 | 44.84 | 44.74 |
|  | *9* | 5.92 | 5.75 | 4.87 | 5.47 | 5.33 | 4.79 | 4.85 | 4.31 | 5.62 | 6.07 | 5.91 | 5.30 |
| Sub-9 | *1* | 22.21 | 24.98 | 24.24 | 25.59 | 24.26 | 26.41 | 24.41 | 20.67 | 21.00 | 30.75 | 23.08 | 24.54 |
| Sub-10 | *1* | 36.72 | 31.76 | 36.00 | 36.82 | 36.32 | 30.39 | 31.58 | 32.62 | 27.02 | 34.76 | 36.74 | 33.40 |
|  | *2* | 23.65 | 22.89 | 21.40 | 22.76 | 21.89 | 22.84 | 22.60 | 23.28 | 24.12 | 22.07 | 23.04 | 22.69 |
|  | *3* | 10.47 | 10.07 | 11.15 | 10.29 | 10.44 | 11.20 | 10.05 | 11.07 | 10.88 | 11.46 | 9.34 | 10.60 |
| Sub-11 | *1* | 35.85 | 42.06 | 43.15 | 35.41 | 39.74 | 42.19 | 39.77 | 37.72 | 40.39 | 42.37 | 43.42 | 40.62 |
|  | *2* | 13.22 | 11.54 | 9.47 | 11.27 | 11.70 | 10.24 | 11.38 | 10.24 | 12.97 | 10.05 | 9.67 | 10.85 |
|  | *3* | 5.95 | 5.63 | 3.91 | 2.88 | 2.82 | 2.88 | 5.79 | 4.89 | 2.69 | 2.95 | 2.92 | 3.74 |
|  | *4* | 14.36 | 7.25 | 7.73 | 5.89 | 7.59 | 7.99 | 6.55 | 8.89 | 8.67 | 9.07 | 7.65 | 7.73 |
|  | *5* | 11.18 | 8.59 | 6.77 | 8.97 | 8.48 | 7.45 | 8.03 | 6.43 | 7.60 | 7.25 | 4.22 | 7.38 |
|  | *6* | 49.65 | 34.05 | 32.29 | 34.55 | 33.91 | 37.87 | 34.55 | 36.49 | 33.20 | 33.43 | 34.86 | 34.52 |
|  | *7* | 22.58 | 12.16 | 10.79 | 13.41 | 9.61 | 10.65 | 10.13 | 11.96 | 13.31 | 13.60 | 10.83 | 11.65 |
|  | *8* | 10.35 | 5.91 | 7.18 | 6.88 | 7.89 | 6.73 | 9.02 | 7.41 | 9.45 | 9.13 | 7.55 | 7.72 |
| Sub-12 | *1* | 8.55 | 5.01 | 3.16 | 6.80 | 4.20 | 4.84 | 3.94 | 6.23 | 4.24 | 3.92 | 3.73 | 4.61 |
| Sub-13 | *1* | 4.81 | 5.94 | 6.40 | 6.59 | 4.58 | 4.77 | 6.87 | 5.27 | 5.71 | 5.14 | 4.62 | 5.59 |
|  | *2* | 11.59 | 6.65 | 8.70 | 8.12 | 6.51 | 6.83 | 6.73 | 6.90 | 8.55 | 5.88 | 8.78 | 7.37 |
|  | *3* | 16.51 | 9.64 | 12.91 | 12.69 | 8.24 | 10.96 | 8.22 | 7.96 | 10.40 | 8.64 | 10.90 | 10.06 |
|  | *4* | 123.68 | 104.06 | 104.20 | 104.81 | 103.03 | 104.21 | 103.45 | 104.81 | 106.24 | 103.98 | 105.02 | 104.38 |
|  | *5* | 66.51 | 44.35 | 44.45 | 43.93 | 45.40 | 46.49 | 45.36 | 45.01 | 43.30 | 44.81 | 42.80 | 44.59 |
|  | *6* | 17.13 | 14.39 | 12.04 | 13.07 | 14.05 | 14.15 | 12.82 | 11.90 | 14.87 | 17.10 | 13.19 | 13.76 |
|  | *7* | 23.58 | 9.15 | 16.81 | 10.63 | 11.76 | 11.08 | 9.66 | 12.07 | 13.71 | 13.03 | 15.80 | 12.37 |
|  | *8* | 12.16 | 6.99 | 6.67 | 6.63 | 6.63 | 6.89 | 5.69 | 4.57 | 5.81 | 7.45 | 5.76 | 6.31 |
|  | *9* | 4.06 | 1.68 | 2.96 | 3.07 | 2.43 | 2.37 | 3.19 | 2.34 | 2.79 | 3.36 | 2.62 | 2.68 |
|  | *10* | 21.38 | 16.13 | 15.05 | 16.87 | 14.19 | 16.81 | 15.35 | 16.54 | 14.50 | 15.87 | 16.81 | 15.81 |
|  | *11* | 5.81 | 1.93 | 3.14 | 2.71 | 2.40 | 3.50 | 2.21 | 2.26 | 2.33 | 2.97 | 0.24 | 2.37 |
|  | *12* | 34.40 | 21.88 | 23.57 | 25.27 | 28.12 | 24.86 | 24.48 | 24.01 | 25.21 | 26.62 | 23.77 | 24.78 |
|  | *13* | 26.95 | 17.52 | 18.41 | 19.85 | 16.20 | 15.85 | 17.80 | 15.57 | 16.09 | 15.54 | 17.33 | 17.02 |
| Sub-14 | *1* | 7.81 | 3.74 | 2.90 | 3.04 | 2.54 | 3.07 | 2.85 | 3.35 | 4.37 | 3.61 | 2.68 | 3.22 |
|  | *2* | 4.43 | 2.38 | 2.10 | 2.16 | 1.69 | 2.01 | 2.02 | 2.16 | 2.00 | 2.08 | 1.86 | 2.05 |
|  | *3* | 16.03 | 19.13 | 10.52 | 13.41 | 9.91 | 15.23 | 14.10 | 10.16 | 14.67 | 12.15 | 18.18 | 13.75 |
|  | *4* | 180.06 | 331.33 | 332.33 | 332.33 | 331.44 | 328.81 | 331.03 | 328.02 | 332.85 | 330.29 | 329.60 | 330.80 |
|  | *5* | 4.62 | 2.82 | 6.42 | 3.25 | 6.29 | 2.16 | 1.94 | 3.19 | 6.66 | 5.87 | 3.55 | 4.22 |
|  | *6* | 4.17 | 5.86 | 6.13 | 2.99 | 6.05 | 5.89 | 5.76 | 6.36 | 5.77 | 5.38 | 5.73 | 5.59 |
|  | *7* | 238.80 | 210.84 | 225.28 | 229.61 | 226.20 | 230.41 | 217.36 | 219.22 | 222.47 | 224.12 | 231.68 | 223.72 |
|  | *8* | 60.38 | 60.92 | 64.41 | 66.35 | 67.58 | 57.18 | 66.49 | 66.13 | 66.61 | 61.45 | 62.18 | 63.93 |
|  | *9* | 6.75 | 2.56 | 3.72 | 2.71 | 2.26 | 5.78 | 6.65 | 2.69 | 2.92 | 6.91 | 6.23 | 4.24 |
|  | *10* | 5.62 | 2.73 | 7.77 | 2.95 | 2.93 | 2.99 | 2.61 | 6.09 | 7.35 | 7.85 | 7.14 | 5.04 |
|  | *11* | 13.21 | 15.40 | 13.86 | 13.76 | 14.20 | 15.06 | 13.46 | 14.81 | 14.76 | 15.40 | 14.40 | 14.51 |
|  | *12* | 15.81 | 12.39 | 13.18 | 12.82 | 11.82 | 12.42 | 12.76 | 12.60 | 12.97 | 12.88 | 11.91 | 12.58 |
|  | *13* | 15.11 | 9.23 | 9.10 | 8.62 | 9.35 | 8.56 | 8.16 | 8.89 | 8.61 | 9.53 | 8.78 | 8.88 |
| Sub-15 | *1* | 257.04 | 250.39 | 245.50 | 252.05 | 244.25 | 238.18 | 244.83 | 251.11 | 249.02 | 247.99 | 248.01 | 247.13 |
| Sub-16 | *1* | 10.00 | 9.66 | 9.33 | 9.86 | 8.21 | 11.84 | 6.92 | 9.97 | 9.65 | 6.89 | 7.74 | 9.01 |
|  | *2* | 2.77 | 1.66 | 1.11 | 0.84 | 0.85 | 3.10 | 1.19 | 0.97 | 0.67 | 0.80 | 0.72 | 1.19 |
|  | *3* | 5.80 | 5.83 | 5.94 | 5.21 | 5.26 | 4.27 | 4.54 | 4.42 | 4.20 | 5.21 | 4.90 | 4.98 |
|  | *4* | 6.75 | 6.47 | 5.30 | 4.78 | 5.82 | 5.31 | 5.24 | 4.39 | 5.19 | 5.17 | 5.89 | 5.36 |

**Supplementary Figure 1:**

**Supplementary Figure 1.** Correlation between motion amplitude and difference in SUV_mean_ between gated and ungated scans. The motion amplitude was estimated by the overall movement of the centroid of each lesion in the three imaging axes (X, Y and Z).

**Supplementary Figure 2:**

**Supplementary Figure 2.** Correlation between motion amplitude and difference in SUV_max_ between gated and ungated scans. The motion amplitude was estimated by the overall movement of the centroid of each lesion in the three imaging axes (X, Y and Z).
